# Circulating Adipokines, Tissue Receptor Expression, and Body Composition: Clinical Relevance and Disease Stage in Epithelial Ovarian Carcinoma

**DOI:** 10.3390/cancers18162712

**Published:** 2026-08-21

**Authors:** Lizeth Montserrat Aguilar-Vazquez, Gabriela Nohemi Espinoza-de-León, Ricardo Misael Alemán-Montes, Diego Alberto Morales-Soto, Jose Ramon Lopez-Lopez, Benjamín González-Amézquita, Raquel Villegas-Pacheco, Aldo Antonio Alcaraz-Wong, Iris Monserrat Llamas-Covarrubias, Erika Martínez-López, Adriana Aguilar-Lemarroy, Luis Felipe Jave-Suárez

**Affiliations:** 1 Programa de Doctorado en Ciencias en Biología Molecular en Medicina, Centro Universitario de Ciencias de la Salud, Universidad de Guadalajara, Guadalajara 44340, Mexico; lizeth.aguilar@alumnos.udg.mx; 2 División de Inmunología, Centro de Investigación Biomédica de Occidente (CIBO), Instituto Mexicano del Seguro Social (IMSS), Guadalajara 44340, Mexico; gabriela.espinoza6107@alumnos.udg.mx (G.N.E.-d.-L.); ricardo.aleman8398@alumnos.udg.mx (R.M.A.-M.); 3 Departamento de Anatomía Patológica, División de Auxiliares del Diagnóstico y Tratamiento, Centro Médico Nacional de Occidente, Instituto Mexicano del Seguro Social (IMSS), Guadalajara 44340, Mexico; alberto.morales7912@alumno.udg.mx (D.A.M.-S.); ramon.lopez1705@alumno.udg.mx (J.R.L.-L.); aldo.alcaraz@imss.gob.mx (A.A.A.-W.); 4 Unidad Médica de Alta Especialidad, Hospital de Ginecología y Obstetricia, Centro Médico Nacional de Occidente, Instituto Mexicano del Seguro Social (IMSS), Guadalajara 44340, Mexico; benggy_09@hotmail.com (B.G.-A.); raquel.villegaspa@imss.gob.mx (R.V.-P.); 5 Instituto de Nutrigenética y Nutrigenómica Traslacional, Centro Universitario de Ciencias de la Salud, Universidad de Guadalajara, Guadalajara 44340, Mexico; iris.llamas@academicos.udg.mx (I.M.L.-C.); erika.martinez@academicos.udg.mx (E.M.-L.); 6 Unidad de Investigación Epidemiológica y en Servicios de Salud, Instituto Mexicano del Seguro Social (IMSS), Guadalajara 44340, Mexico

**Keywords:** epithelial ovarian carcinoma, leptin, resistin, adiponectin, adipsin, adipokine receptors, body composition

## Abstract

Background/Objectives: Nutritional status and adipose tissue-derived mediators have been implicated in the development and clinical characteristics of epithelial ovarian carcinoma; however, studies integrating circulating adipokines, adipokine receptor expression, and nutritional status remain limited. This study aimed to evaluate circulating adipokine concentrations, adipokine receptor expression, nutritional status parameters, and their associations with clinically relevant features and disease stage in women with epithelial ovarian carcinoma. Methods: A total of 151 women were included, comprising 43 patients with epithelial ovarian carcinoma, 89 with benign ovarian tumors, and 19 healthy controls. The nutritional status parameters included body composition, dietary intake, and serum glucose concentration. Circulating adipokines were quantified by ELISA, whereas adipokine receptor expression was evaluated by automated immunohistochemistry in patients with available tumor tissue. Associations with clinicopathological variables were assessed using correlation analyses and multivariable regression models. Results: Compared with women with benign ovarian tumors, women with epithelial ovarian carcinoma had significantly lower circulating leptin concentrations, whereas their circulating resistin concentrations were significantly higher than in healthy controls. In multivariable analysis, lower leptin and higher resistin concentrations were associated with clinical disease classification, while lower leptin was also associated with advanced-stage disease. Visceral fat was associated with clinical disease classification. In addition, AdipoR1 expression was significantly more frequent in malignant than in benign ovarian tumors. Conclusions: Circulating leptin and resistin, visceral fat, and AdipoR1 expression were associated with clinically relevant characteristics across the study groups. Together, these findings provide a more comprehensive characterization of the metabolic and inflammatory alterations associated with epithelial ovarian carcinoma and may contribute to the identification of potential clinically relevant markers.

## Simple Summary

Epithelial ovarian carcinoma is often diagnosed at advanced stages, highlighting the need for biomarkers that improve our understanding of disease pathophysiology. Increasing evidence suggests that nutritional status and molecules produced by adipose tissue may influence tumor behavior, although their clinical relevance remains unclear. In this study, we evaluated circulating adipokines, the expression of their receptors in ovarian tissue, and parameters related to nutritional status in women with epithelial ovarian carcinoma, benign ovarian tumors, and healthy controls. We found that lower leptin concentrations, higher resistin concentrations, visceral fat, and more frequent AdipoR1 expression were associated with clinically relevant differences across the study groups. By integrating metabolic, nutritional, and tissue-related characteristics within the same patient cohort, this study provides a more comprehensive characterization of metabolic alterations associated with epithelial ovarian carcinoma and highlights potential markers for future clinical research.

## 1. Introduction

Ovarian cancer (OC) is among the deadliest gynecological malignancies worldwide. According to GLOBOCAN 2024 estimates, OC accounted for approximately 330,731 new cases and 203,850 deaths, ranking as the eighth most frequently diagnosed cancer and the eighth leading cause of cancer-related death among women globally. Among gynecological malignancies, it represents the fourth most common cancer and the third leading cause of cancer-related mortality [1]. Histologically, OC comprises epithelial, germ cell, and sex cord-stromal tumors, of which epithelial ovarian carcinoma (EOC) represents the most frequent and clinically relevant subtype because of its high mortality and biological heterogeneity [2]. Despite advances in surgical techniques and systemic therapies, the prognosis of EOC remains poor, largely because most patients are diagnosed at advanced stages due to the nonspecific nature of early symptoms and the lack of effective screening strategies [3,4]. These challenges highlight the need to improve the biological understanding of EOC and to identify clinically relevant biomarkers associated with disease status and clinical stage.

Beyond tumor-related characteristics, increasing evidence suggests that host-related factors, particularly nutritional status, also influence the development and clinical behavior of EOC [5]. Overweight and obesity (OB) have been associated with an increased risk of OC; however, this relationship remains unclear across studies and populations [6,7]. Nevertheless, in addition to its potential role in disease development, nutritional status at diagnosis has emerged as an important determinant of treatment response, treatment-related toxicity, and survival in patients with cancer, including those with EOC [8,9,10]. Traditionally, nutritional status has been assessed using body mass index (BMI); however, BMI alone does not adequately reflect alterations in body composition. Patients with similar BMI values may present marked differences in skeletal muscle mass, visceral fat, or even sarcopenic obesity, conditions that have been associated with poorer clinical outcomes in women with OC [5,11]. Likewise, dietary intake has received considerably less attention despite its potential influence on metabolic homeostasis and systemic inflammation [5]. Therefore, a comprehensive nutritional assessment integrating dietary intake and body composition may provide a more accurate characterization. One of the biological mechanisms proposed to link nutritional status with tumor development and tumor aggressiveness involves the endocrine activity of adipose tissue. In addition to its role in energy storage, adipose tissue is recognized as an active endocrine organ capable of secreting numerous bioactive molecules, collectively known as adipokines (ADPs) [12].

ADPs are a group of bioactive molecules secreted predominantly by adipose tissue that regulate energy homeostasis, inflammation, insulin sensitivity, angiogenesis, and immune responses [13,14]. Beyond their physiological functions, accumulating evidence suggests that ADPs participate in multiple processes involved in cancer development and progression, including cell proliferation, invasion, angiogenesis, and metastatic dissemination [13,14]. However, the biological effects of ADPs appear to be heterogeneous. While some ADPs have been associated with tumor-promoting activities, others have been proposed to exert antitumor effects through the modulation of pathways involved in cancer development and progression [13,14].

The biological activity of ADPs is mediated through their interaction with specific membrane receptors expressed in target tissues. Consequently, both the concentration of circulating ADPs and receptor expression may influence the biological effects of these molecules within the tumor microenvironment. However, compared with that of circulating ADPs, the evaluation of ADP receptor expression in EOC remains limited, and its potential clinical relevance has not been fully elucidated [15,16].

Clinical studies evaluating circulating ADPs in women with EOC have produced inconsistent findings. Although leptin (LPN) and adiponectin (APN) have been the most frequently investigated circulating ADPs, evidence regarding resistin (RST) and adipsin (ADS) remains limited. Moreover, previous studies have generally evaluated these biomarkers independently and have rarely integrated circulating ADPs with dietary intake, body composition, tissue receptor expression, and clinicopathological characteristics within the same patient cohort [17,18].

Therefore, the aim of this study was to evaluate the concentrations of circulating ADPs, the expression of ADP receptors, and nutritional status parameters, and to investigate their associations with clinically relevant features and clinical stage in patients with EOC.

## 2. Materials and Methods

### 2.1. Study Design, Participants, and Clinical Data Collection

An observational, analytical, cross-sectional study was conducted at the Unidad Médica de Alta Especialidad: Hospital de Ginecología y Obstetricia (HGO) of the Centro Médico Nacional de Occidente (CMNO) of the Instituto Mexicano del Seguro Social (IMSS), Mexico, and the Centro de Investigación Biomédica de Occidente (CIBO), IMSS. The study was approved by the Comité Nacional de Investigación Científica (CNIC) of the IMSS, under the registration number R-2025-785-007 approved on 22 January 2025. A total of 151 women were included in this study. Among these participants, 43 were diagnosed with EOC, 89 had benign tumors, and 19 were healthy donors without cancer and served as the control group. All participants involved in the study read and signed an informed consent form before any procedures were performed. Participants with ovarian tumors were evaluated prior to obtaining a definitive histopathological diagnosis. Women presenting with a tumor of uncertain or unknown behavior of the ovary and scheduled for diagnostic or exploratory surgery at the HGO-CMNO during the study period were consecutively screened for eligibility and invited to participate before surgery. All potentially eligible patients were approached for recruitment. The inclusion criteria for the study were as follows: age between 18 and 75 years, no prior systemic oncological treatment, no autoimmune diseases, and no previous diagnosis of any type of cancer. Following surgery and histopathological confirmation, a post-enrollment exclusion criterion was applied. Cases in which the final diagnosis indicated a nonepithelial ovarian malignancy or a nongynecological pathology were excluded from the final analysis. Tumors were classified as benign or malignant on the basis of a comprehensive pathological evaluation that included macroscopic examination, hematoxylin and eosin (H&E) staining, and microscopic assessment. All histopathological evaluations were performed by board-certified pathologists in the Division of Diagnostic and Treatment Support Services, CMNO, IMSS. In the control group, participants were required to have no evidence of ovarian tumors, as confirmed by pelvic ultrasound. Additionally, they had no gynecological diseases and no autoimmune disorders. Healthy controls were recruited through public advertisements, including social media announcements and printed posters, and eligibility was confirmed through clinical evaluation and pelvic ultrasound. Sociodemographic characteristics, along with personal and family medical histories, lifestyle-related factors, and gynecological and obstetric histories, were collected through a structured clinical interview conducted at enrollment. Additional clinical and pathological data, including tumor size, histological subtype, and, when applicable, International Federation of Gynecology and Obstetrics (FIGO) stage and other tumor-related characteristics, were obtained from a review of medical records.

### 2.2. Serum Sample Collection

For patients with ovarian tumors, a single peripheral blood sample was collected prior to surgery during the preoperative fasting period and before the initiation of any oncological treatment. Blood samples from healthy controls were collected under fasting conditions at enrollment. Blood samples were obtained via venipuncture of the cubital vein using the BD Vacutainer^®^ system(Becton, Dickinson and Company, Franklin Lakes, NJ, USA) and collected in red-top tubes made of polyethylene terephthalate, without anticoagulants. The samples were then centrifuged at 2500 rpm for 10 min at 20 °C. Following centrifugation, the serum was separated into 200 μL aliquots and stored at −80 °C until further analysis.

### 2.3. Tissue Sample Collection and Histopathological Confirmation

Tumor tissue samples were obtained from patients included in the serum analyses, allowing the direct evaluation of circulating analytes and tissue receptor expression within the same individual. Tissue analyses were performed exclusively in patients with benign ovarian tumors and EOC; no tissue samples, biopsies, or surgical specimens were obtained from healthy controls.

Following surgical resection and definitive histopathological diagnosis performed at the Division of Diagnostic and Treatment Support Services, CMNO, IMSS, we requested the corresponding formalin-fixed paraffin-embedded (FFPE) tissue blocks from the pathology archives. Particular care was taken to retrieve the exact FFPE block used for the original diagnostic evaluation, ensuring direct correspondence between the pathological diagnosis and the tissue analyses performed in this study.

Serial sections were obtained from each FFPE block. One section was stained with H&E and reviewed by a board-certified pathologist to confirm that the selected tissue block corresponded to the histopathological diagnosis reported in the original pathology record. Additional sections were used for subsequent immunohistochemical analyses. After sectioning, all FFPE blocks were returned to the pathology archives.

Although tissue samples were requested from all eligible patients with ovarian tumors, not every FFPE block was available for retrieval at the time of the study. In some cases, tissue blocks were temporarily unavailable because they had been removed from the pathology archives for routine clinical purposes, including external review or additional diagnostic evaluation. Consequently, immunohistochemical analyses were performed only for cases in which the corresponding tissue block could be recovered from the pathology archives. A total of 52 benign and 35 malignant ovarian tissue samples were available for immunohistochemistry (IHC) analyses.

### 2.4. Nutritional and Body Composition Assessment

Several nutritional status components were evaluated prior to surgery and during the same visit when the blood samples were collected. All nutritional assessments were performed by a trained nutritionist and included anthropometric measurements, body composition analysis, dietary assessment, and serum glucose determination.

Body composition was assessed using a TANITA FITSCAN BC-601FS bioelectrical impedance analyzer (Tanita Corporation, Tokyo, Japan). This device provided measurements of body weight, BMI, total body fat percentage, muscle mass, and visceral fat index. Height was measured using a SECA stadiometer (SECA GmbH, Hamburg, Germany), while body circumferences were measured with a SECA measuring tape (SECA GmbH, Hamburg, Germany). All anthropometric measurements were performed in accordance with the guidelines set by the International Society for the Advancement of Kinanthropometry (ISAK). In patients with a documented report of ascites, body weight, body composition, and circumferential measurements were reassessed after surgery to evaluate the potential influence of ascitic fluid on these measurements.

Dietary intake was assessed using two 24 h dietary recalls, including one weekday and one weekend day, both of which referred to the period prior to hospitalization. During the interviews, participants were guided in the estimation of food portions and beverage consumption using standardized household measures to improve the accuracy of dietary reporting. Dietary recalls were analyzed using the AZ nutrition software (version 20) to estimate total energy intake (kcal), carbohydrate, protein, and lipid consumption, as well as selected micronutrients.

Serum glucose concentrations were determined using an automated VITROS^®^ 350 analyzer (Ortho Clinical Diagnostics, Raritan, NJ, USA) and an enzymatic glucose reagent based on the glucose oxidase/peroxidase method, according to the manufacturer’s instructions.

### 2.5. Determination of Circulating Adipokines

Serum concentrations of APN, LPN, ADS, and RST were determined by enzyme-linked immunosorbent assay (ELISA) using the following commercially available kits from R&D Systems (Minneapolis, MN, USA): Human Adiponectin/Acrp30 Quantikine ELISA Kit (Cat. No. DRP300), Human leptin Quantikine ELISA Kit (Cat. No. DLP00), Human Complement Factor D (Adipsin) ELISA Kit (Cat. No. DFD00), and Human Resistin Quantikine ELISA Kit (Cat. No. DRSN00). All serum samples were analyzed in duplicate, and all assays were performed according to the manufacturer’s instructions. Serum aliquots used for ELISA measurements were thawed only once, and no repeated freeze–thaw cycles were performed prior to analysis. The assay sensitivities were <7.8 pg/mL for LPN, 0.246 ng/mL for APN, 0.013 ng/mL for ADS, and 0.026 ng/mL for RST. According to the manufacturer, intra-assay coefficients of variation did not exceed 6.8%, whereas inter-assay coefficients of variation did not exceed 9.2% for any of the ELISAs used in this study. A colorimetric detection method was used, and the absorbance was measured using a Multiskan™ Sky Microplate Spectrophotometer (Thermo Fisher Scientific, Waltham, MA, USA) at 450 nm with wavelength correction at 540 nm. ADP concentrations were calculated from standard curves generated for each assay and expressed according to the units specified by the manufacturer.

Previously determined serum IL-6 concentrations from the same study cohort were retrieved to perform exploratory correlation analyses with circulating ADPs. IL-6 was quantified using the LEGENDplex™ Custom Human Panel (Cat. No. 900007431, BioLegend, San Diego, CA, USA) according to the manufacturer’s instructions. Samples were analyzed by flow cytometry using a CytoFLEX V4-B4-R3 flow cytometer (Beckman Coulter Inc., Brea, CA, USA), and concentrations were calculated using the LEGENDplex™ Data Analysis Software Suite (BioLegend, San Diego, CA, USA), which is available online (https://legendplex.qognit.com/user/login, accessed on 9 September 2025). Since IL-6 concentrations have been reported previously [19], only correlation analyses involving IL-6 are presented in the current study.

### 2.6. Determination of Serum CA125 and HE4 Levels and Calculation of the ROMA Index

Serum concentrations of cancer antigen 125 (CA125) and human epididymis protein 4 (HE4) were determined by ELISA using the following commercially available kits from R&D Systems (Minneapolis, MN, USA): Human CA125/MUC16 Quantikine ELISA Kit (Cat. No. DCA125) and Human HE4/WFDC2 Quantikine ELISA Kit (Cat. No. DHE400). Serum samples were analyzed in duplicate, and all assays were performed according to the manufacturer’s instructions.

The risk of ovarian malignancy algorithm (ROMA) index was calculated using serum CA125 and HE4 concentrations together with menopausal status (premenopausal or postmenopausal), according to previously published equations [20].

For premenopausal women:

PI = −12.0 + 2.38 × ln(HE4) + 0.0626 × ln(CA125)

For postmenopausal women:

PI = −8.09 + 1.04 × ln(HE4) + 0.732 × ln(CA125)

Predicted Probability (PP)

PP = [e^PI^/(1 + e^PI^)] × 100

### 2.7. Tissue Sectioning and Hematoxylin and Eosin Staining

After the FFPE tissue blocks were retrieved, serial 4 μm thick sections were obtained using a microtome (Cat. No. RM2125 RTS, Leica Biosystems, Wetzlar, Germany). The tissue sections were floated on a distilled water bath at 43 °C and mounted onto positively charged Apex adhesive slides (Cat. No. 3800080E, Leica Biosystems, Wetzlar, Germany). The slides were subsequently dried in an oven at 50 °C for 1 h and stored in slide boxes (Cat. No. HS-15994G, Heathrow Scientific, Vernon Hills, IL, USA) until further processing.

For H&E staining, the slides were incubated at 55 °C to melt residual paraffin, followed by deparaffinization in two xylene baths. The tissue sections were subsequently rehydrated through a graded ethanol series (100%, 100%, 96%, and 80%) and rinsed in distilled water. Slides were stained with hematoxylin for 4 min, washed in distilled water, differentiated in 96% ethanol, and counterstained with eosin for 90 s. After staining, the sections were dehydrated through graded ethanol solutions (80%, 96%, 100% and 100%), cleared in two xylene baths, and mounted using CV Ultra mounting medium (Cat. No. 14070937981, Leica Biosystems, Wetzlar, Germany). The slides were coverslipped and allowed to dry overnight at room temperature.

### 2.8. Immunohistochemistry Assays

ADP receptor expression was evaluated by IHC using a Bond Max automated stainer (Cat. No. 49.0051, Leica Biosystems, Wetzlar, Germany). The tissue sections were placed in instrument trays, covered with Leica covertiles, and processed according to the manufacturer’s instructions. Antigen retrieval was performed for 20 min using either BOND Epitope Retrieval Solution 1 (ER1: a pH 6 citrate-based buffer, Cat. No. AR9961, Leica Biosystems, Wetzlar, Germany) or BOND Epitope Retrieval Solution 2 (ER2: a pH 8 EDTA-based buffer; Cat. No. AR9640, Leica Biosystems, Wetzlar, Germany).

The following primary antibodies, validated for IHC and obtained from GeneTex (Irvine, CA, USA), were used: AdipoR1 (Adiponectin Receptor 1 antibody [C2C3], C-term; Cat. No. GTX104770; dilution 1:250; ER2), AdipoR2 (Adiponectin Receptor 2 antibody; Cat. No. GTX04314; dilution 1:400; ER1), and LepR (Leptin Receptor antibody [GT1233]; Cat. No. GTX02830; dilution 1:500; ER1). All primary antibodies were diluted using Bond™ Primary Antibody Diluent (Cat. No. AR9352, Leica Biosystems, Wetzlar, Germany). Prior to the analysis of patient samples, staining conditions for each antibody were optimized using control tissues with known expression patterns. The final antibody dilutions reported above were selected after evaluating the range of dilutions recommended by the manufacturer and identifying the conditions that provided the best balance between staining intensity and background signal. During the optimization process, negative control sections were processed without the primary antibody, using only Bond™ Primary Antibody Diluent (Cat. No. AR9352, Leica Biosystems, Wetzlar, Germany), to confirm the absence of nonspecific staining attributable to the staining procedure. The tissue sections were incubated with primary antibodies for 15 min at room temperature. Immunoreactivity was detected using the BOND Polymer Refine Detection system (Cat. No. DS9800, Leica Biosystems, Wetzlar, Germany). After completion of the automated staining process in the Bond Max system, the slides were manually dehydrated through graded ethanol solutions (95% and absolute ethanol) and cleared in xylene (two immersions of 15 s each). Slides were subsequently mounted and coverslipped using CV Ultra mounting medium (Leica Biosystems).

### 2.9. Evaluation of Adipokine Receptor Expression

IHC staining was independently evaluated by three pathologists from the Division of Diagnostic and Treatment Support Services, CMNO, IMSS. Receptor expression was assessed using the Allred scoring system, which combines the proportion of positively stained cells and staining intensity to generate a total score ranging from 0 to 8. The final score assigned to each sample was established by consensus among the three pathologists. The proportion score was assigned according to the percentage of positive cells as follows: 0 (0%), 1 (<1%), 2 (1–10%), 3 (11–33%), 4 (34–66%), and 5 (>66%). Staining intensity was classified as 0 (none), 1 (weak), 2 (moderate), or 3 (strong). The final Allred score was obtained by summing the proportion and intensity scores. Samples with total scores of 0–2 were classified as negative, whereas samples with scores ranging from 3 to 8 were classified as positive [21].

### 2.10. Statistical Analysis

Statistical analyses were performed using RStudio (version 2024.12.1+563, Posit Software, Boston, MA, USA). Data distribution was assessed using the Shapiro–Wilk and Kolmogorov–Smirnov tests, and the homogeneity of variance was evaluated using Levene’s test. Categorical variables were expressed as frequencies and percentages, whereas continuous variables were expressed as means ± standard deviations or medians (interquartile ranges), according to their distribution. Comparisons between two groups were performed using Student’s *t*-test for normally distributed variables or the Mann–Whitney U test for nonnormally distributed variables. Comparisons among three or more groups were conducted using one-way analysis of variance (ANOVA), Welch’s one-way ANOVA, or the Kruskal–Wallis test, as appropriate. When significant differences were detected, post hoc analyses were performed using Dunn’s test with Bonferroni correction or the Games-Howell test, as appropriate. Categorical variables were compared using the chi-square test or Fisher’s exact test, as appropriate. Associations between continuous variables were evaluated using Spearman’s correlation coefficient. Regression analyses were performed to further investigate associations among clinical, nutritional, biochemical, and pathological variables. Linear regression models were used for continuous outcomes, binary logistic regression models for dichotomous outcomes, and ordinal logistic regression models for ordered categorical outcomes. Multivariable models were adjusted for potential confounding factors when appropriate. Covariates were selected based on their relevance and on differences and associations observed in the descriptive and correlation analyses. Continuous predictors included in the logistic regression models were standardized, and odds ratios (ORs) and 95% confidence intervals (CIs) were expressed per one-standard-deviation increase. The proportional odds assumption was assessed using the Brant test, and a partial proportional odds model was additionally performed to estimate threshold-specific effects for variables showing non-proportional effects. Multicollinearity was evaluated using variance inflation factors (VIFs), and goodness of fit of the binary logistic regression models was assessed using the Hosmer-Lemeshow test. Regression analyses were performed using complete cases, without imputation of missing data. An a priori sample-size calculation was performed during the study design based on the expected proportion of OC in the Mexican population. For all tests, a two-sided alpha of 0.05 was used to establish statistical significance.

## 3. Results

### 3.1. Clinical and Tumor Characteristics of the Study Population

A total of 151 participants were included in the study, comprising 43 patients with EOC, 89 patients with benign ovarian tumors, and 19 healthy controls. The clinical characteristics and serum biomarkers of the study population are summarized in Table 1. Patients with EOC were significantly older than those in the benign tumor group (*p* < 0.0001). Similarly, serum CA125 and HE4 concentrations, as well as the ROMA index, were significantly greater in the EOC group than in both the benign tumor and control groups (all *p* < 0.0001). The frequency of postmenopausal women was significantly higher in the EOC group than in the benign tumor group (*p* < 0.001). In contrast, parity, age at menarche, diabetes mellitus, oral contraceptive use, and smoking status did not differ significantly among the study groups.

Among women with ovarian tumors, those in the EOC group presented significantly larger tumors than those with benign tumors (Table 2). High-grade serous carcinoma was the most frequent histological subtype of EOC, followed by endometrioid, clear cell, and mucinous carcinomas, whereas low-grade serous carcinoma represented the least frequent subtype. A similar distribution of disease stage was observed between early-stage (FIGO I-II) and advanced-stage (FIGO III-IV) EOC patients. FIGO stage information was unavailable for two patients. In the benign tumor group, endometriomas, serous cystadenomas, and mature teratomas were the most common histological subtypes.

### 3.2. Components of Nutritional Status

The components of nutritional status are summarized in Table 3. Significant differences in BMI and visceral fat index were observed among the study groups. Both variables were lower in controls than in women with benign tumors and EOC. No significant differences were observed for total body fat percentage, muscle mass, mid-upper arm circumference (MUAC), or serum glucose concentration.

Regarding dietary intake, total energy consumption tended to be lower in the control and EOC groups; however, the difference did not reach statistical significance. Similarly, the percentage contribution of carbohydrates, lipids, and proteins to total energy intake did not differ among the groups.

Differences were observed in selected micronutrients. Women with benign tumors and EOC had lower intakes of saturated fatty acids and folate than those in the control group. Similarly, non-heme iron intake was lower in both tumor groups than in the control group. Total iron intake also differed among the study groups, with lower intake in the control group than in the benign tumor group. The intake of additional dietary components and micronutrients did not differ significantly among the groups and is summarized in Supplementary Table S1.

The frequency of self-reported early satiety differed among the study groups and was more frequently reported in women with ovarian tumors than in controls.

### 3.3. Circulating Adipokine Levels According to Study Group and Disease Stage

**Figure 1:**
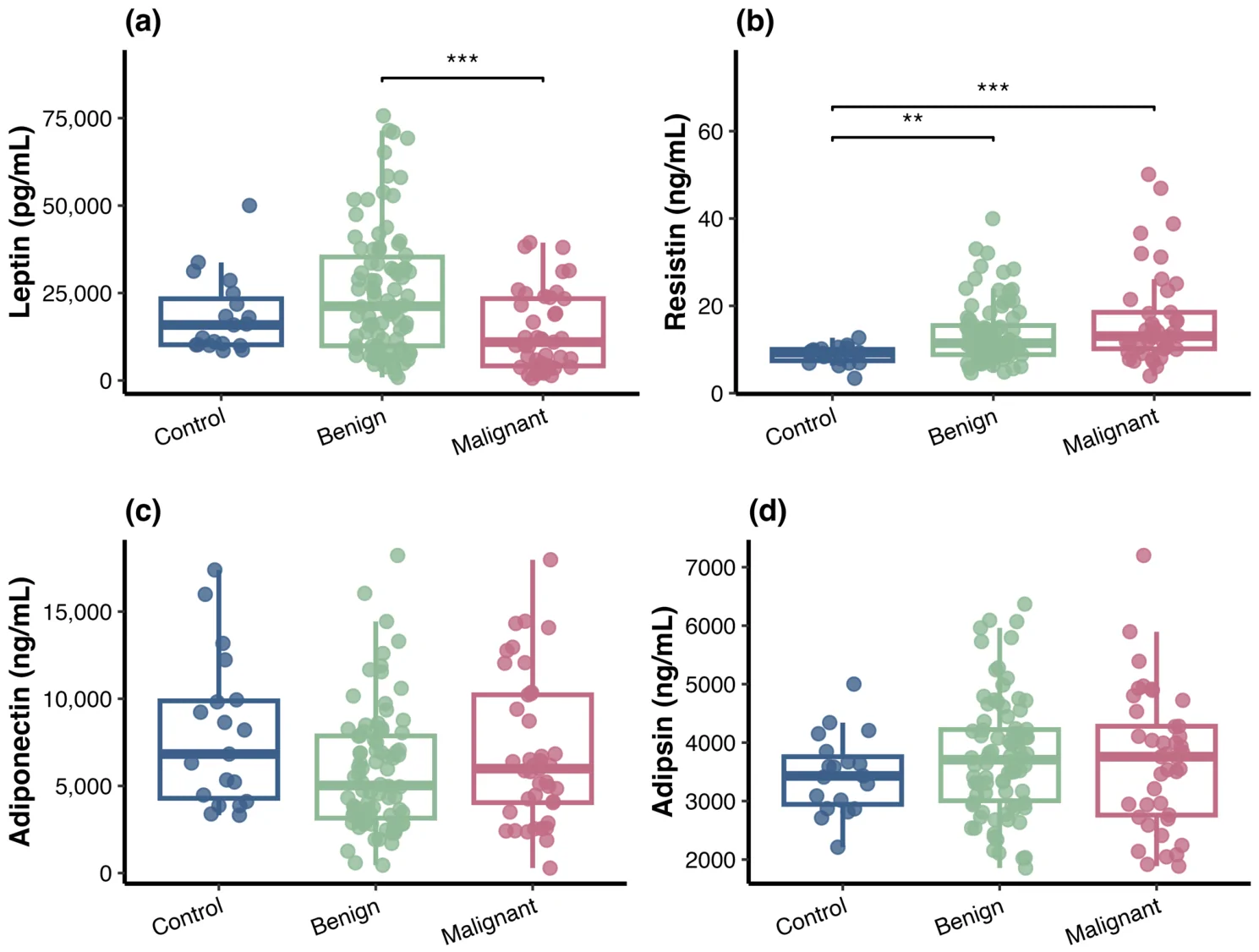
Comparison of circulating adipokines among study groups. (**a**) Leptin, (**b**) Resistin, (**c**) Adiponectin, and (**d**) Adipsin serum concentrations. Box plots show the median and interquartile range, with individual observations overlaid as points. Statistical significance is indicated by ** *p* < 0.01, and *** *p* < 0.001. Differences among groups were evaluated using the Kruskal–Wallis test followed by Dunn’s multiple comparisons test with Bonferroni correction.

Serum concentrations of ADPs were measured by ELISA and compared among women with EOC, benign ovarian tumors, and controls (Figure 1). LPN concentrations were significantly lower in patients with EOC than in women with benign tumors (10,964 [4125–23,564] vs. 21,032 [9831–33,573] pg/mL, *p* < 0.001). The median LPN concentration in control group was 15,868 (10,210–23,366) pg/mL and did not differ significantly from that in either tumor group.

In contrast, RST concentrations were significantly higher in women with EOC than in controls (13.1 [10.3–19.9] vs. 9.2 [7.4–10.0] ng/mL, *p* < 0.001). RST levels were also higher in women with benign tumors than in controls (11.4 [8.8–15.4] vs. 9.2 [7.4–10.0] ng/mL, *p* < 0.01). No significant differences were observed among the groups for APN or ADS.

**Figure 2:**
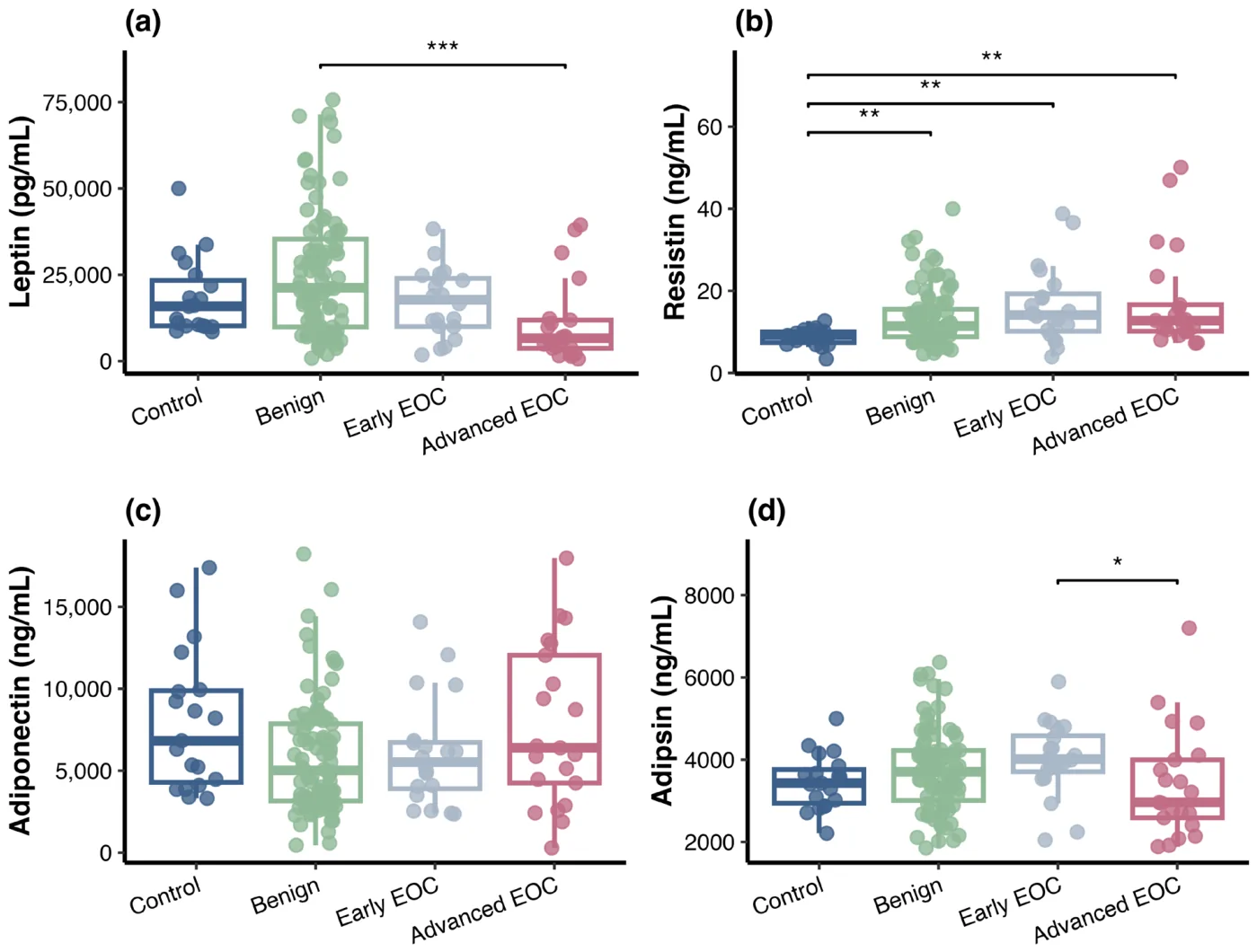
Comparison of circulating adipokine concentrations according to study group and FIGO stage. (**a**) Leptin, (**b**) Resistin, (**c**) Adiponectin, and (**d**) Adipsin serum concentrations. Box plots show the median and interquartile range, with individual observations overlaid as points. Statistical significance is indicated by * *p* < 0.05, ** *p* < 0.01, and *** *p* < 0.001. Differences among groups were evaluated using the Kruskal–Wallis test followed by Dunn’s multiple comparisons test with Bonferroni correction.

To further evaluate the relationship between ADPs and disease status and stage, serum ADP concentrations were compared among controls, benign tumors, early-stage EOC, and advanced-stage EOC (Figure 2). LPN concentrations were significantly lower in women with advanced-stage EOC than in those with benign tumors (6628 [3750–11,876] vs. 21,032 [9831–33,573] pg/mL, *p* < 0.001). RST levels were significantly higher in both early-stage and advanced-stage EOC patients than in controls (14.1 [10.1–19.2] and 12.7 [10.1–16.6] vs. 9.2 [7.4–10.0] ng/mL, respectively; both *p* < 0.01). In addition, RST concentrations remained significantly higher in women with benign tumors than in controls (11.4 [8.8–15.4] vs. 9.2 [7.4–10.0] ng/mL, *p* < 0.01). In contrast, ADS concentrations were significantly lower in women with advanced-stage EOC than in those with early-stage disease (2961 [2589–3994] vs. 4015 [3707–4580] ng/mL, *p* < 0.05). The magnitude of the observed group differences was further assessed using η^2^[H] effect size estimates derived from the Kruskal–Wallis analyses (Supplementary Table S2). LPN and RST showed moderate effect sizes across both study groups and disease-status categories, whereas APN and ADS showed small effect sizes.

### 3.4. Expression of Adipokine Receptors in Ovarian Tissue

The results of the IHC analysis of ADP receptor expression are summarized in Table 4. A total of 35 malignant and 52 benign ovarian tissue samples were available for analysis.

AdipoR1 expression was significantly more frequent in malignant tissues than in benign tissues (88.6% [31/35] vs. 61.5% [32/52], *p* < 0.01). In contrast, AdipoR2 expression was infrequent in both groups, with positive staining observed in only 2.9% (1/35) of malignant and 5.8% (3/52) of benign tissues. Similarly, LepR expression was detected in 37.1% (13/35) of malignant tissues and 23.1% (12/52) of benign tissues, with no significant difference between groups.

**Figure 3:**
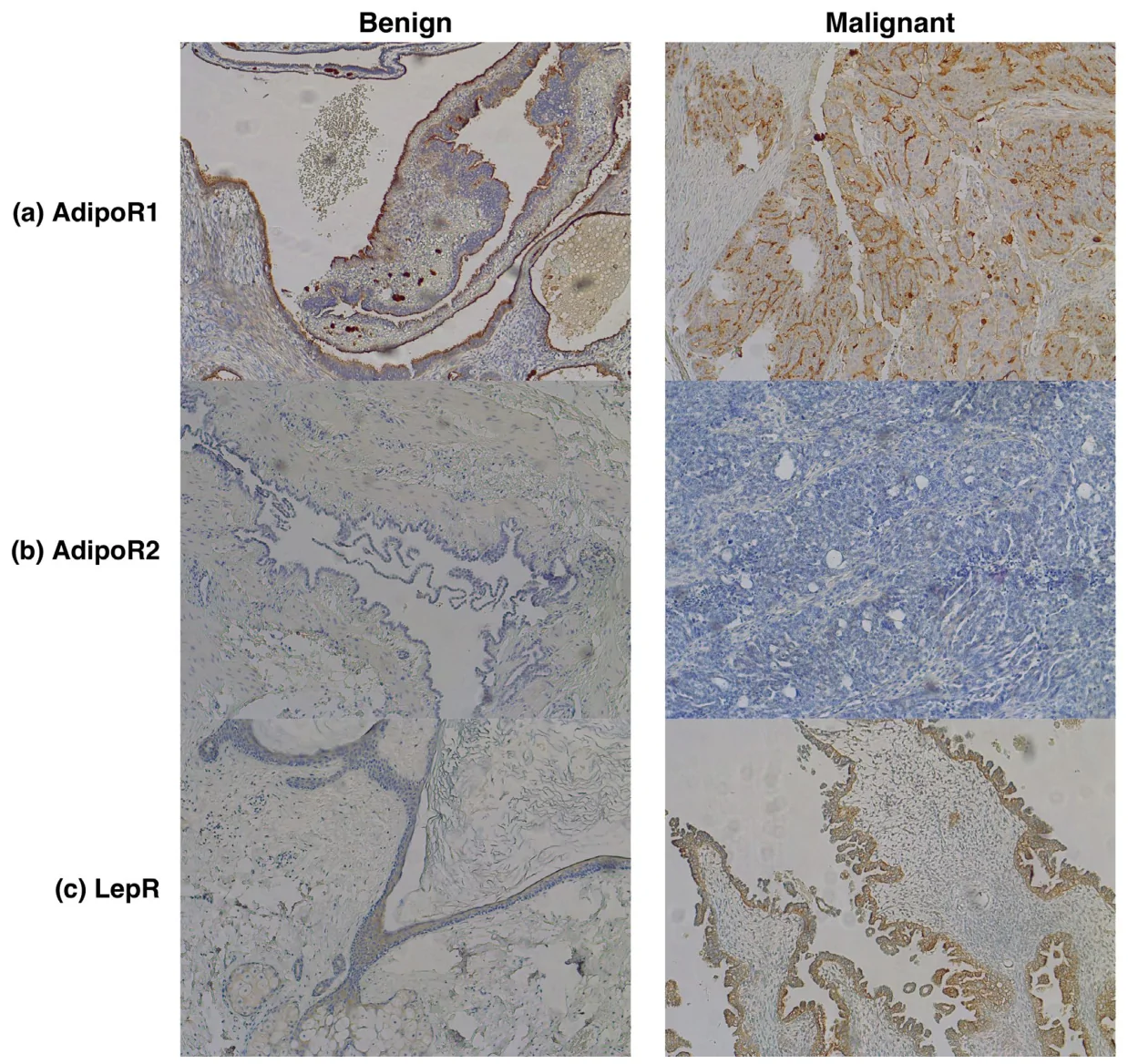
Representative immunohistochemical images showing the expression of adipokine receptors in benign and malignant ovarian tumors. Representative images of (**a**) positive AdipoR1 expression in a benign serous cystadenoma (located in the upper portion of the tissue section) and a high-grade serous carcinoma; (**b**) negative AdipoR2 expression in a benign serous cystadenoma and a high-grade serous carcinoma; and (**c**) positive LepR expression in a benign mature teratoma and a high-grade serous carcinoma. All images were acquired using a 10× objective.

Representative IHC staining patterns for AdipoR1, AdipoR2, and LepR in benign and malignant ovarian tissues are shown in Figure 3. The figure includes representative images from six patients (three benign and three malignant), illustrating positive and negative receptor expression with varying staining intensities across the different histological samples. Additional representative cases are provided in Supplementary Figure S1.

### 3.5. Associations Between Adipokines, Components of Nutritional Status, and Clinical Variables

**Figure 4:**
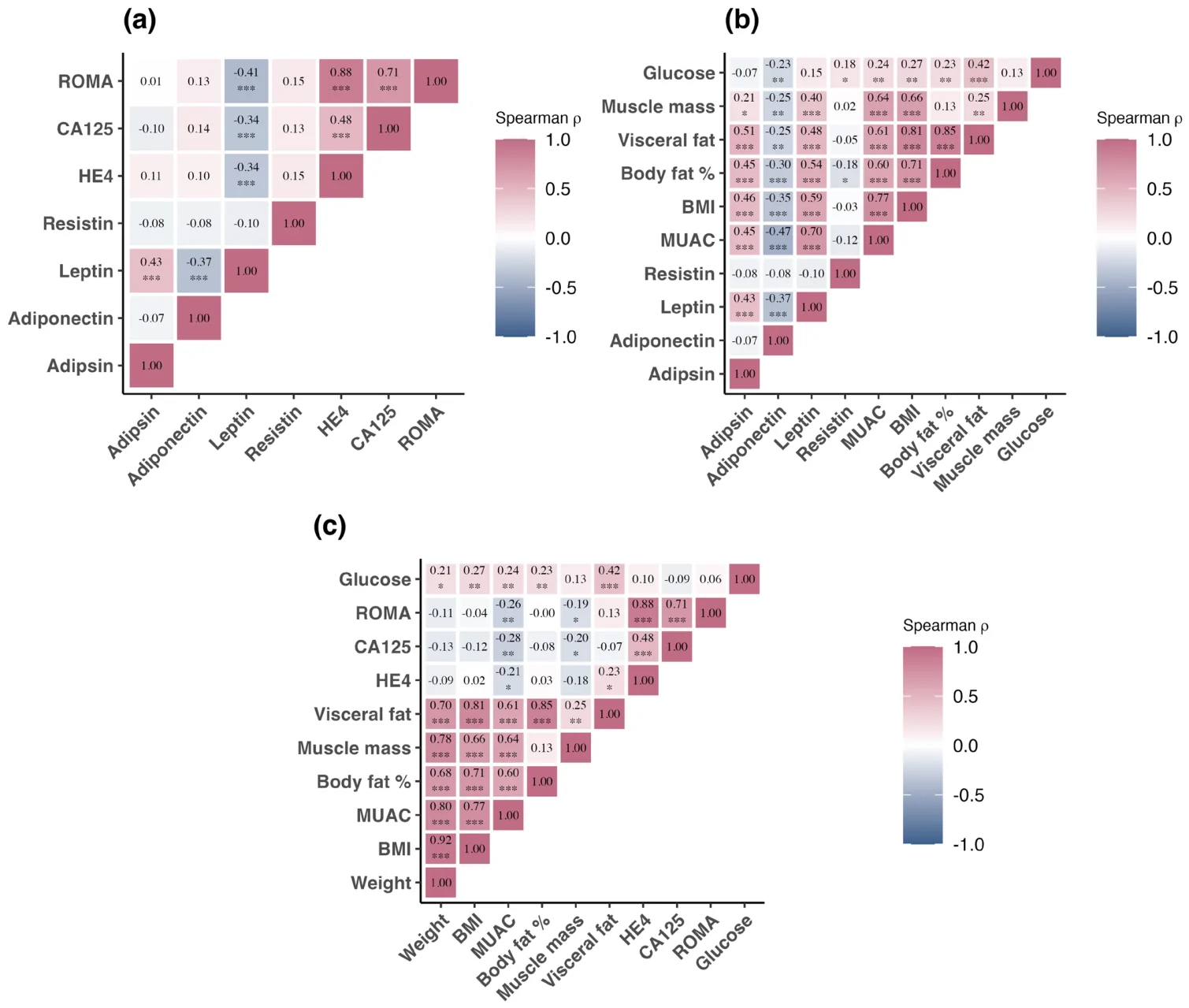
Spearman correlation (ρ) analysis of adipokines, nutritional status components, and ovarian cancer biomarkers. (**a**) Correlations between circulating adipokines and ovarian cancer biomarkers. (**b**) Correlations between circulating adipokines and components of nutritional status. (**c**) Correlations between components of nutritional status and ovarian cancer biomarkers. The values indicate Spearman’s correlation coefficients. Asterisks denote statistical significance: * *p* < 0.05, ** *p* < 0.01, and *** *p* < 0.001.

Spearman correlation analyses were performed to explore the associations between circulating ADPs, components of nutritional status, and clinical variables (Figure 4).

First, correlations between serum ADPs concentrations and OC biomarkers were evaluated (Figure 4a). LPN showed moderate inverse correlations with the ROMA index (ρ = −0.41; *p* < 0.001), CA125 concentration (ρ = −0.34; *p* < 0.001), and HE4 concentration (ρ = −0.34; *p* < 0.001). In contrast, APN, ADS, and RST were not associated with these biomarkers.

Next, the relationships between ADPs and components of nutritional status, including body composition parameters, serum glucose concentrations, and the ADPs themselves, were examined (Figure 4b). ADS was positively correlated with BMI, MUAC, total body fat percentage, visceral fat, and muscle mass. In addition, ADS concentration showed a moderate positive correlation with LPN concentration (ρ = 0.43; *p* < 0.001). APN was inversely associated with BMI, MUAC, total body fat percentage, visceral fat index, muscle mass, serum glucose levels. In addition, APN showed moderate inverse correlation with LPN concentrations (ρ = −0.37; *p* < 0.001). LPN was positively correlated with BMI, MUAC, total body fat percentage, visceral fat index, and muscle mass. RST was not significantly associated with body composition parameters or with the other ADPs; however, a weak positive correlation was observed with serum glucose concentrations (ρ = 0.18; *p* < 0.05). Correlations between dietary intake variables and circulating ADPs were also explored. Only a few weak correlations reached statistical significance (Supplementary Figure S2).

Finally, associations between components of nutritional status and OC biomarkers were evaluated (Figure 4c). MUAC was inversely correlated with all three OC biomarkers, including CA125, HE4, and the ROMA index. In contrast, muscle mass showed inverse correlations with CA125 and the ROMA index, but not with HE4. In addition, HE4 showed a weak positive correlation with the visceral fat index (ρ = 0.23; *p* < 0.05).

An exploratory analysis was also performed to evaluate the relationship between circulating ADPs and IL-6 levels in women with EOC. A moderate inverse correlation was observed between LPN and IL-6; however, this association did not reach statistical significance (ρ= −0.33, *p* = 0.067). RST showed a moderate positive correlation with IL-6 (ρ = 0.36; *p* < 0.05; Supplementary Figure S3).

Correlations between circulating ADPs and age were also evaluated (Supplementary Table S3). In the overall cohort, only ADS showed a weak positive correlation with age (ρ = 0.21; *p* < 0.05), whereas APN, LPN, and RST were not significantly associated with age. In the EOC subgroup, APN showed a moderate positive correlation with age (ρ = 0.35; *p* < 0.05), while LPN showed a moderate inverse correlation (ρ = −0.30; *p* < 0.05). No significant associations were observed between age and ADS or RST in women with EOC.

### 3.6. Regression Analyses

**Figure 5:**
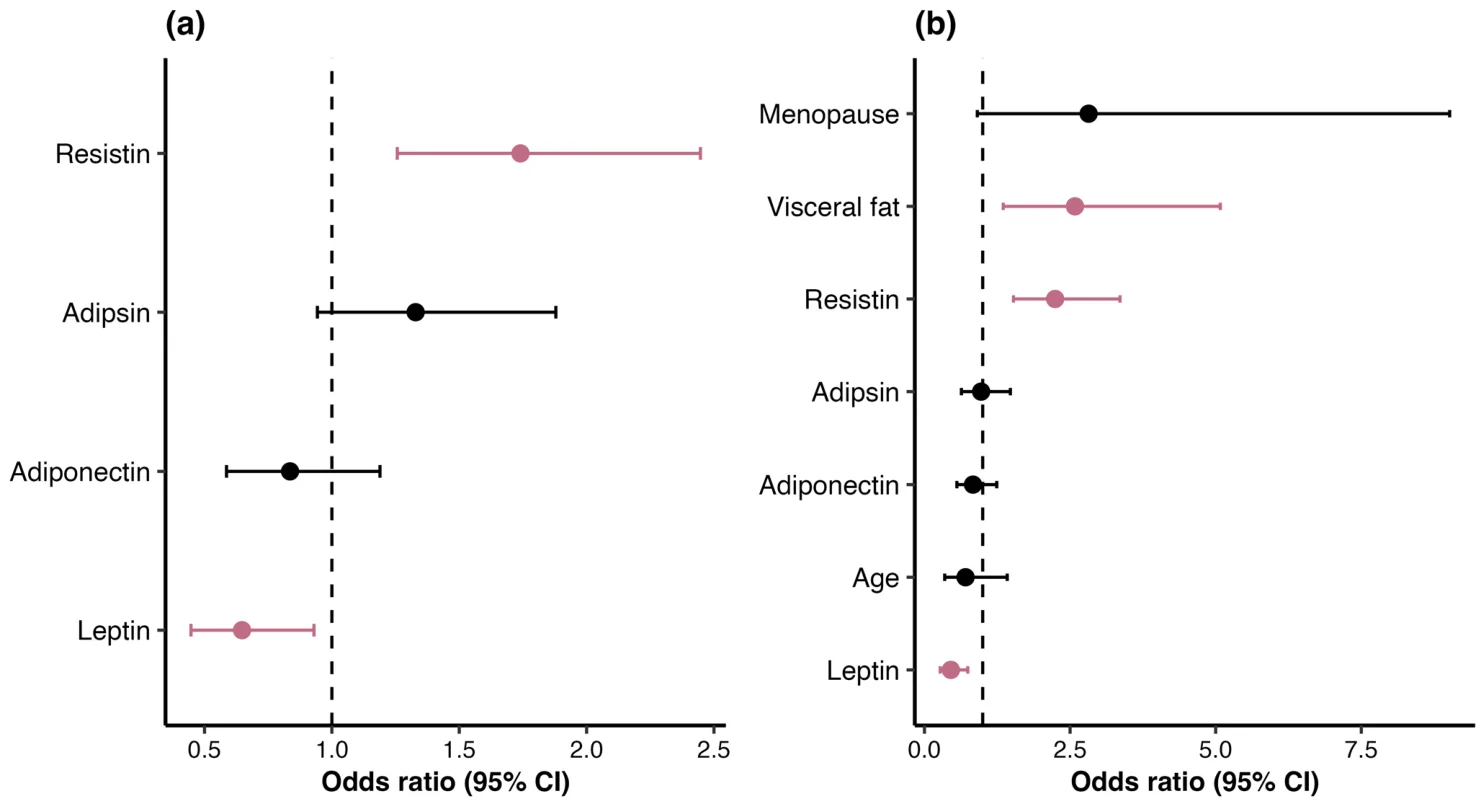
Ordinal logistic regression analysis of associations between adipokines and the ordered clinical outcome. Odds ratios (ORs) and 95% confidence intervals obtained from ordinal logistic regression models. (**a**) Unadjusted model including adipokines only. (**b**) Model adjusted for visceral fat, age, and menopausal status. The ordered outcome was defined as Control < Benign ovarian tumor < Early-stage EOC < Advanced-stage EOC. Continuous predictors were standardized, and ORs are expressed per one-standard-deviation increase. Variables with 95% CIs not crossing 1 (dashed line) were considered statistically significant (pink dots).

Ordinal logistic regression models were used to evaluate whether circulating ADP concentrations were independently associated with an ordered clinical outcome (Figure 5). Disease status was specified as an ordered outcome (Control < Benign < Early EOC < Advanced EOC). This predefined ordering represents a clinically meaningful gradient of disease severity but does not imply the biological progression or natural history of a single disease. ORs are expressed per one standard deviation increase in the corresponding variable. Because the outcome was modeled as an ordered variable, ORs greater than 1 indicate an increased likelihood of belonging to a higher category within the predefined clinical ordering, whereas an OR less than 1 indicates a decreased likelihood.

In the unadjusted model, higher RST levels were associated with higher categories within the predefined clinical ordering (OR = 1.70; 95% CI: 1.22–2.40; *p* < 0.01), whereas LPN was inversely associated with the ordered outcome (OR = 0.64; 95% CI: 0.44–0.93; *p* < 0.05). To determine whether these associations were independent of adiposity, age, and menopausal status, an additional model was adjusted for these variables. After adjustment, RST remained positively associated with higher categories within the predefined clinical ordering (OR = 2.24; 95% CI: 1.53–3.36; *p* < 0.001), while LPN remained inversely associated with the ordered outcome (OR = 0.45; 95% CI: 0.27–0.74; *p* < 0.01). Visceral fat index was also independently associated with higher categories within the predefined clinical ordering (OR = 2.58; 95% CI: 1.35–5.08; *p* < 0.01) (Figure 5b). Complete results of the adjusted model are presented in Supplementary Table S4, which includes both the primary model adjusted for visceral fat and a sensitivity model adjusted for BMI. Multicollinearity diagnostics are provided in Supplementary Table S5. The proportional odds assumption was not fully met in the ordinal logistic regression models; therefore, a partial proportional odds model was additionally performed as a sensitivity analysis, allowing the effects of LPN, RST, and visceral fat to vary across cumulative outcome thresholds. This analysis showed threshold-dependent associations for these variables, with significant associations observed at selected cumulative outcome thresholds. Complete estimates are provided in Supplementary Table S4.

**Figure 6:**
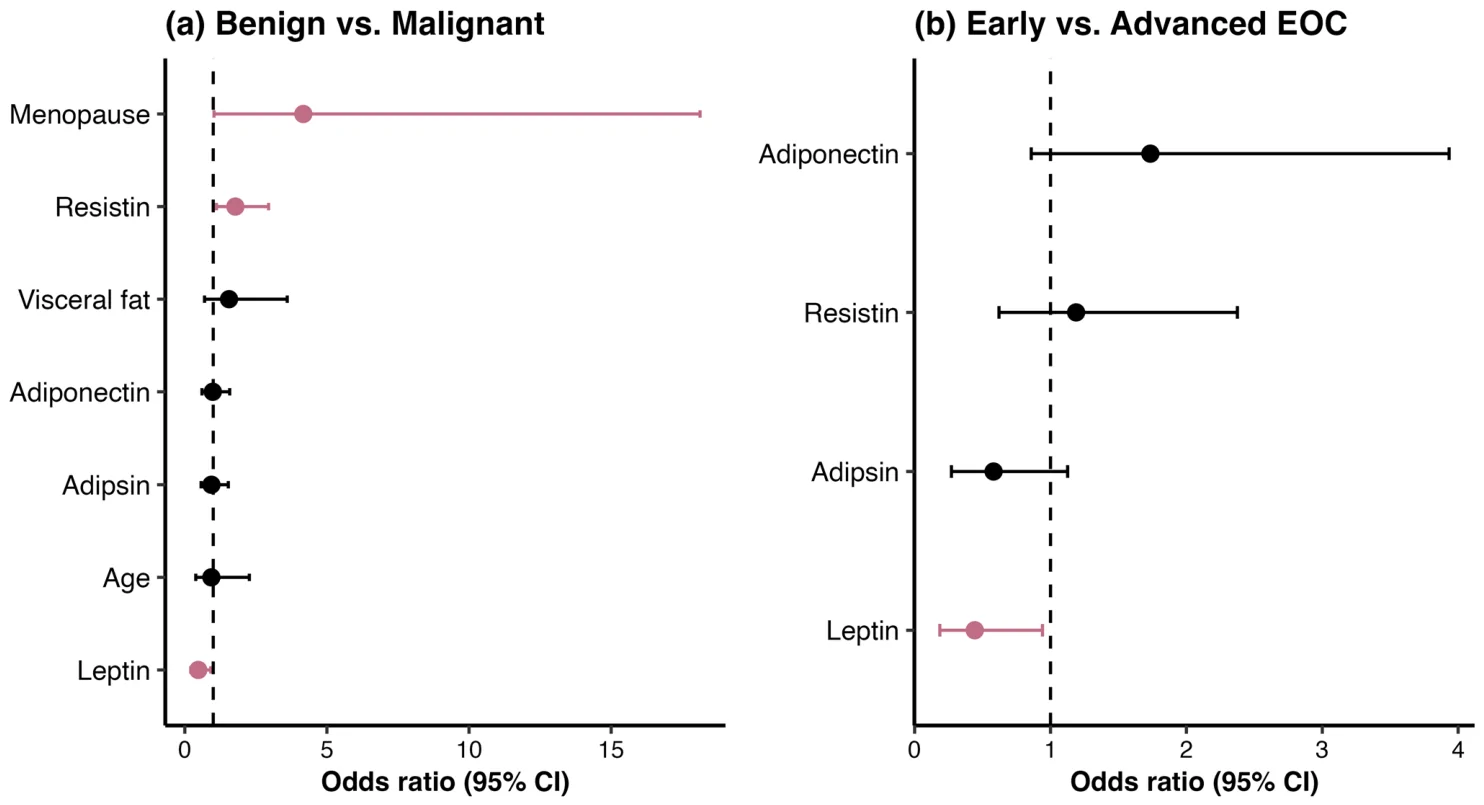
Binary logistic regression analyses of associations between adipokines, visceral fat, ovarian tumor malignancy, and epithelial ovarian carcinoma stage. Odds ratios (OR) and 95% confidence intervals obtained from logistic regression models. (**a**) Multivariable model discriminating benign from malignant ovarian tumors (n = 132) adjusted for visceral fat, age, and menopausal status. (**b**) Separate binary logistic regression models for each adipokine discriminating advanced from early-stage EOC (n = 41), adjusted for age and menopausal status. For visual clarity, only adipokine estimates are displayed in panel (**b**); complete model estimates, including adjustment covariates, are provided in Supplementary Table S6. Variables with 95% confidence intervals not crossing 1 (dashed line) were considered statistically significant (pink dots).

To complement the ordinal analysis, binary logistic regression models were fitted (Figure 6). In these analyses, lower LPN concentrations and higher RST concentrations were independently associated with malignant versus benign ovarian tumors. Menopausal status was also associated with malignancy, although the estimate showed a wide confidence interval. In contrast to the ordinal model, visceral fat was not independently associated with malignancy in the binary analysis. Among women with EOC, lower LPN concentrations remained independently associated with advanced-stage disease after adjustment for age and menopausal status. The binary models showed adequate goodness of fit according to the Hosmer-Lemeshow test, except for the APN model, although APN itself was not significantly associated with advanced-stage disease. Complete numerical results of the binary logistic regression models are provided in Supplementary Table S6.

### 3.7. Association Between Circulating Adipokines and Receptor Expression

**Figure 7:**
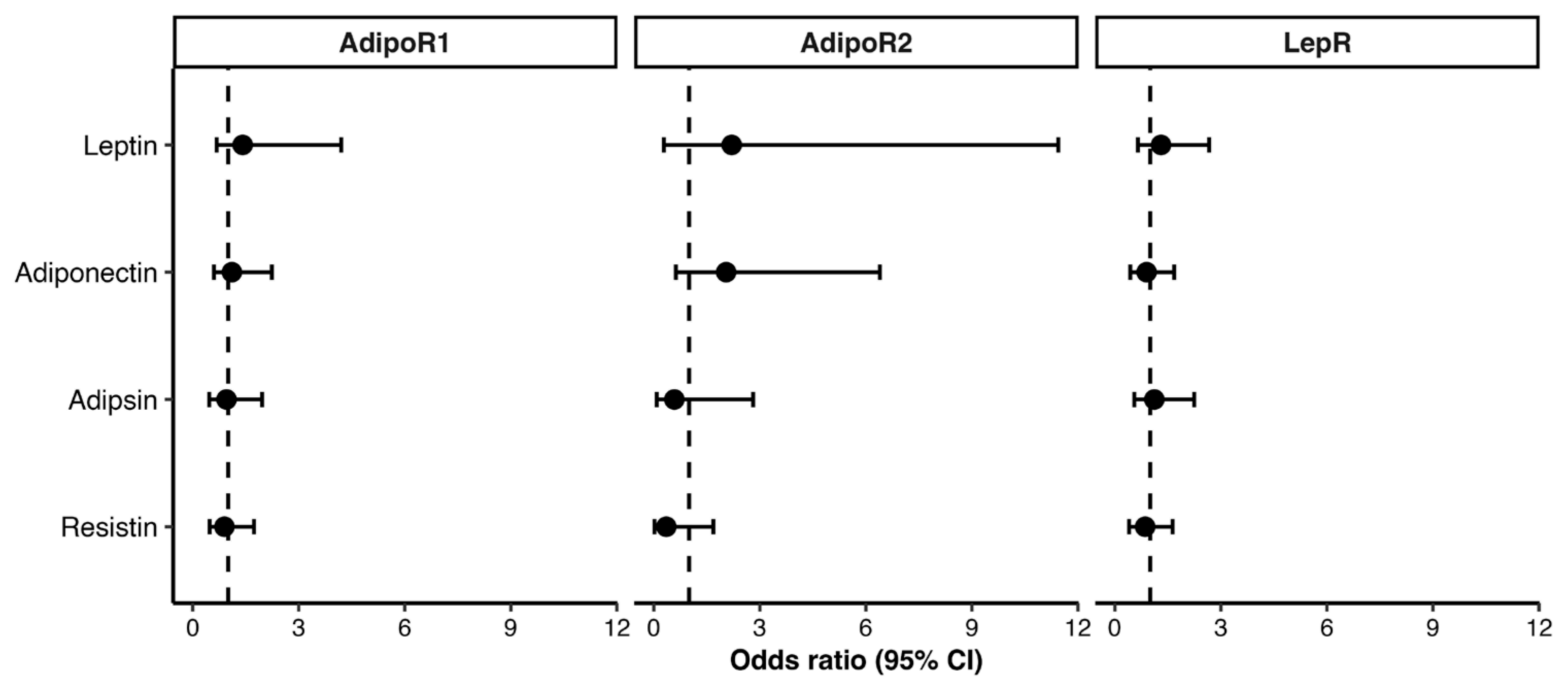
Logistic regression analysis of the associations between circulating adipokines and adipokine receptor expression in the subset of patients with available tissue samples (n = 87; 35 malignant and 52 benign). Odds ratios (OR) and 95% confidence intervals were estimated using binary logistic regression models. Separate models were fitted for LepR, AdipoR1, and AdipoR2 expression. Variables with 95% confidence intervals not crossing 1 (dashed line) were considered statistically significant. Models were fitted using standardized predictor variables, and receptor expression was analyzed as a binary outcome (positive vs. negative), with negative expression used as the reference category.

Finally, binary logistic regression models were used to evaluate the associations between circulating ADP concentrations and ADP receptor expression. No significant associations were observed between circulating LPN, APN, ADS, or RST concentrations and the expression of LepR, AdipoR1, or AdipoR2 (Figure 7).

**Figure 8:**
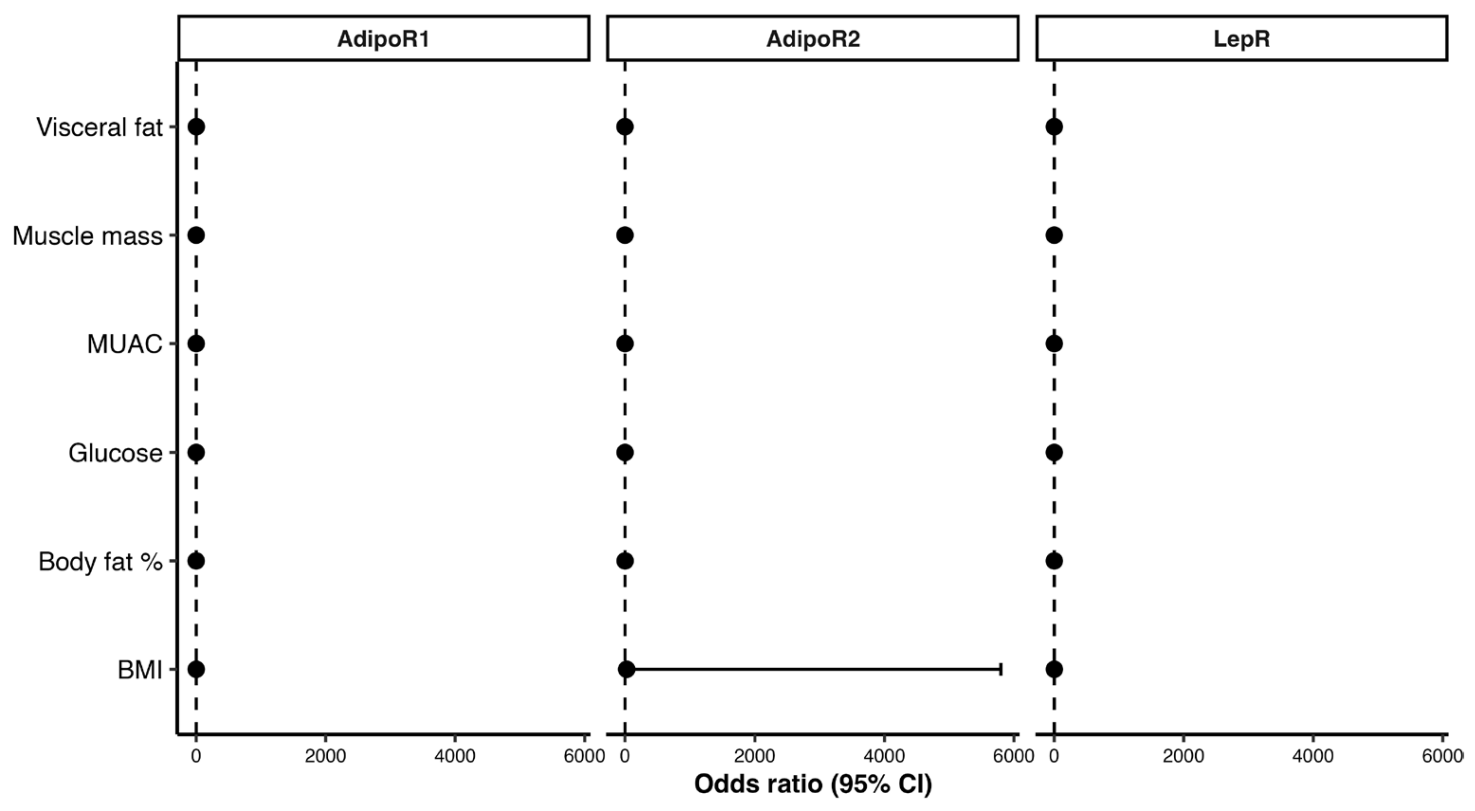
Logistic regression analysis of the associations between components of nutritional status and adipokine receptor expression in the subset of patients with available tissue samples (n = 87; 35 malignant and 52 benign). Odds ratios (ORs) and 95% confidence intervals were estimated using binary logistic regression models. Separate models were fitted for AdipoR1, AdipoR2, and LepR expression. Variables with 95% confidence intervals not crossing 1 were considered statistically significant. Models were fitted using standardized predictor variables, and receptor expression was analyzed as a binary outcome (positive vs. negative), with negative expression used as the reference category.

Additional binary logistic regression models were performed to examine whether components of nutritional status were associated with ADP receptor expression. No significant associations were identified between BMI, total body fat percentage, visceral fat index, muscle mass, MUAC, or fasting glucose concentration, and the expression of AdipoR1, AdipoR2, or LepR (Figure 8).

## 4. Discussion

EOC is the most lethal gynecological malignancy and is characterized by rapid tumor proliferation and frequent diagnosis at advanced stages, all of which contribute to its poor prognosis [22,23]. In recent years, increasing attention has been given to the role of adipose tissue-derived factors, particularly ADPs, in cancer biology [17]. These molecules have been implicated in several processes involved in tumor development and progression. Moreover, altered circulating ADPs concentrations have been reported in OC [17]. In the present study, a panel of circulating ADPs was evaluated together with multiple components of nutritional status, including body composition parameters beyond BMI, dietary intake variables, and metabolic markers. This comprehensive approach was adopted because BMI alone may not adequately reflect alterations in body composition or metabolic status that frequently accompany cancer. Moreover, nutritional assessment at the time of diagnosis is particularly relevant in OC, as the disease may be accompanied by changes in adiposity, muscle mass, dietary intake, and metabolic homeostasis. The expression of selected ADP receptors was also assessed in tumor tissue. In addition, associations among circulating ADPs, nutritional status components, clinical stage, and established OC biomarkers, including CA125, HE4, and the ROMA index, were investigated.

The clinical characteristics of the study population were generally consistent with the known epidemiological profile of EOC. Compared with women with benign ovarian tumors and healthy controls, women with EOC were older, and they were more frequently postmenopausal than women with benign ovarian tumors [24,25]. As expected, serum CA125, HE4, and ROMA index values were significantly elevated in the malignant group [26,27]. Overall, these findings are in agreement with previous reports describing the clinical presentation of OC.

Regarding components of nutritional status, women with ovarian tumors exhibited higher BMI and visceral fat index values than healthy controls. In the adjusted ordinal model, visceral fat was associated with the predefined clinical ordering. However, because the proportional odds assumption was not fully met, the partial proportional odds model indicated that this association was threshold-dependent: visceral fat was significantly associated with Control|Benign and Benign|Early EOC cumulative threshold, but not with the Early EOC|Advanced EOC threshold. In a recent international perception-based survey, healthcare professionals involved in the care of women with OC reported overweight/obesity and sarcopenia among the nutritional problems most frequently observed at the time of diagnosis [28]. Consistent with this notion, previous studies have demonstrated the prognostic relevance of body composition in OC. Ham et al., reported that a high visceral fat-to-muscle ratio was an independent predictor of poor overall survival [29], whereas Cuello et al., reported that body composition phenotypes, unlike BMI, were associated with survival outcomes, with high adiposity and sarcopenia linked to poor prognosis [30]. Similarly, Torres et al., reported that alterations in body composition were independently associated with poor overall survival in women with OC [31].

The association observed between visceral fat and disease status categories highlights the potential relevance of visceral adiposity in this context. Visceral fat is a metabolically active organ and has been associated with systemic inflammation, altered ADP secretion, and metabolic dysregulation, processes that have been implicated in cancer biology [32,33,34]; however, these mechanisms were not directly evaluated in the present study. In addition, a weak positive correlation between visceral fat index and HE4 concentrations was observed in this study. Although studies evaluating visceral fat and OC biomarkers remain limited, Behrouzi et al. reported positive associations between adiposity-related parameters and circulating HE4 concentrations [35].

In terms of dietary intake, the higher prevalence of early satiety among women with ovarian tumors may influence food choices and contribute to the differences in nutrient intake observed in this study. Early satiety, anorexia, and reduced food intake are common manifestations of OC and may contribute to nutritional deterioration during disease progression [36].

Among the ADPs evaluated, LPN was associated with multiple disease-related variables. Women with EOC had lower circulating LPN concentrations than women with benign tumors, with the lowest concentrations observed in women with advanced EOC. In addition, LPN was negatively correlated with HE4, CA125, and the ROMA index, while positive correlations were observed with body composition parameters. Furthermore, lower LPN concentrations remained independently associated with higher categories within the predefined clinical ordering after adjustment for visceral fat, age, and menopausal status. These findings are consistent with previous studies reporting reduced circulating LPN concentrations in women with EOC. Jin et al. reported lower plasma LPN concentrations in EOC patients and observed a significant positive correlation between LPN and BMI; however, they found no differences according to clinical stage [37]. Similarly, Hasenburg et al. reported significantly lower plasma LPN concentrations in patients with EOC compared with women with benign ovarian tumors [38]. Grabowski et al. reported lower circulating LPN concentrations in women with EOC than in those with benign ovarian tumors, with the lowest levels observed in advanced disease. In addition, they reported a positive correlation between LPN and BMI and demonstrated a significant increase in circulating LPN concentrations following complete cytoreductive surgery [39]. Similarly, Slomian et al. observed lower LPN concentrations before treatment, followed by a significant increase after chemotherapy, and reported associations between circulating LPN levels, age, and BMI [40]. Interestingly, the reduced circulating LPN concentrations observed in clinical studies contrast with experimental evidence describing protumorigenic effects of LPN signaling in OC. This apparent discrepancy has been previously recognized and may reflect differences between systemic LPN concentrations and the local activity of the LPN-LepR axis [41]. Experimental studies have shown that LPN-LepR signaling can promote OC cell proliferation, survival, and migration through pathways including JAK2/STAT3 [42]. Although previous studies consistently reported reduced circulating LPN concentrations in women with EOC, the clinical and nutritional variables evaluated differed among studies. In the present study, LPN was evaluated together with body composition parameters beyond BMI, dietary intake, fasting glucose concentration, and OC biomarkers, and multivariable analyses were performed. This approach showed that lower circulating LPN concentrations remained independently associated with higher categories within the predefined clinical ordering after adjustment for visceral fat, age, and menopausal status. Notably, this inverse association was also observed in the partial proportional odds and EOC-only analyses, with lower LPN concentrations associated with advanced-stage disease. Among the available studies, Macciò et al. conducted one of the most comprehensive evaluations of circulating LPN in EOC by examining its relationship with disease stage, BMI, inflammatory mediators, and markers of energy metabolism using correlation and multivariable regression analyses [43]. Similar to our findings, they demonstrated that disease stage remained independently associated with circulating LPN concentrations after adjustment for BMI, suggesting that adiposity alone may not fully account for the observed alterations in circulating LPN concentrations. On the basis of these findings, the authors proposed that reduced circulating LPN concentrations may reflect disease-related metabolic alterations driven, at least in part, by systemic inflammation. In the present study, several variables related to this hypothesis were also explored. However, circulating LPN concentrations were not associated with fasting glucose levels or reported energy intake, while a moderate inverse correlation with IL-6 that did not reach statistical significance was observed among women with EOC (Supplementary Figure S2). The inverse correlations observed between circulating LPN concentrations and CA125, HE4, and the ROMA index further indicate an association between lower circulating LPN concentrations and higher levels of established OC biomarkers. Although previous studies have primarily explored the clinical relevance of LPN in ascitic fluid, Lane et al. and Matte et al. reported that LPN-based indices combined with CA125 levels were associated with treatment response and prognosis in women with EOC [44,45]. While our study evaluated circulating rather than ascitic LPN, these findings collectively suggest that LPN may provide complementary clinical information when interpreted alongside established OC biomarkers.

Compared with LPN, clinical studies evaluating circulating RST concentrations in women with EOC remain scarce. Most of the available evidence has focused on RST expression in tumor tissue or on experimental models investigating its biological role. In the present study, circulating RST concentrations were significantly higher in women with EOC than in healthy controls, with higher concentrations observed in both early and advanced stage EOC. Moreover, higher circulating RST concentrations remained independently associated with higher categories within the predefined clinical ordering after adjustment for visceral fat, age, and menopausal status. In the partial proportional odds analysis, this association was observed at selected cumulative thresholds, whereas RST was not independently associated with advanced-stage disease in the EOC-only analysis. Unlike LPN, circulating RST concentrations were not associated with body composition parameters, indicating that the observed associations with the clinical categories were not accompanied by detectable associations with the body composition parameters evaluated in this study. Although clinical evidence regarding circulating RST concentrations in EOC remains limited, Pang et al. evaluated RST expression in ovarian tissue and reported that higher RST expression was associated with higher pathological grade, lymph node metastasis, and poorer survival. Furthermore, RST expression was identified as an independent prognostic factor for overall survival. Although tissue expression cannot be directly compared with circulating concentrations, these findings provide relevant clinical context for the associations observed in the present study [46]. Experimental studies have also described several tumor-related effects of RST in OC. Pang et al. demonstrated that RST promotes angiogenesis through VEGF induction [47], whereas Qiu et al. reported that RST enhances OC cell proliferation, invasion, epithelial–mesenchymal transition, and cisplatin resistance [48]. Although these findings derive from experimental models, they provide biological context for the associations observed in our study between circulating RST concentrations and the predefined clinical categories. In addition, circulating RST concentrations were positively correlated with IL-6 among women with EOC, suggesting a possible relationship between RST and inflammatory processes in EOC. Elevated circulating RST concentrations have likewise been reported in several other malignancies, including colorectal and non-small cell lung cancer, supporting the concept that RST may participate in tumor-promoting pathways beyond OC [49,50]. Nevertheless, additional clinical studies evaluating circulating RST concentrations in women with EOC are needed to better define their clinical relevance.

Previous studies evaluating circulating APN concentrations in women with EOC have yielded inconsistent results. Several authors have reported lower APN concentrations in women with EOC than in controls or benign ovarian tumors [37,40,51,52], whereas others have reported no significant differences in APN concentrations between patient groups [53]. In the present study, circulating APN concentrations did not differ among study groups or across the predefined clinical categories. Instead, APN was significantly inversely correlated with LPN, fasting glucose concentration, and multiple body composition parameters, including BMI, body fat percentage, and visceral adiposity. These associations indicate that circulating APN concentrations were related to several components of metabolic and nutritional status in the present population, despite the absence of significant differences according to disease status. This finding is consistent with the well-established role of APN in energy homeostasis and insulin sensitivity and agrees with previous observations by Stefan et al. who reported an inverse relationship between APN and endogenous glucose production independent of body fat [54].

Evidence regarding circulating ADS concentrations in EOC remains scarce. In the present study, circulating ADS concentrations did not differ between women with EOC, benign ovarian tumors, and healthy controls. However, significantly lower ADS concentrations were observed in women with advanced EOC than in those with early-stage disease. These findings differ from those reported by Periyasamy et al. who reported lower circulating ADS concentrations in women with EOC than in those with benign ovarian tumors [55]. Notably, the influence of nutritional status and body composition on circulating ADS concentrations was not reported in that study, which should be considered when comparing the findings of the two studies. Interestingly, circulating ADS concentrations were positively correlated with multiple body composition parameters. Similar associations have been reported in non-oncological populations, where circulating ADS concentrations increase in parallel with adiposity-related anthropometric measures [56]. Together, these findings may indicate that circulating ADS concentrations are related to body composition in this population, while the lower concentrations observed in advanced compared with early-stage EOC warrant further investigation.

To further characterize the ADP axis in EOC, the present study simultaneously evaluated circulating LPN and APN concentrations together with the tissue expression of their corresponding receptors. AdipoR1 was significantly more frequently expressed in EOC than in benign ovarian tumors, whereas AdipoR2 expression was uncommon in both groups. Evidence regarding APN receptor expression in EOC remains limited. Li et al. reported positive AdipoR1 expression in approximately 70% of EOC specimens, without significant differences compared with normal ovarian tissue. Furthermore, AdipoR1 positivity was inversely associated with advanced FIGO stage and poor tumor differentiation and independently predicted longer progression-free and overall survival, although no association was observed with BMI [57]. In contrast, the present study found that AdipoR1 positivity was significantly more frequent in malignant than in benign ovarian tumors. Similarly, receptor expression was not associated with BMI or other anthropometric parameters. Although these findings cannot be directly compared because different reference tissues were used, both studies showed frequent AdipoR1 expression in EOC, although its biological and clinical relevance remains to be established. Similarly, Chou et al. evaluated APN receptor expression across several malignancies and reported positive AdipoR1 expression in 87.3% of ovarian carcinomas, whereas only 25.4% of cases showed robust AdipoR1 immunoreactivity. In the same study, AdipoR2 positivity was observed in 54.0% of ovarian carcinomas, although robust expression was detected in only 14.3% of cases [58]. Although direct comparison with the present study is limited, both studies showed a higher frequency of AdipoR1 than AdipoR2 in EOC. The biological significance of the higher frequency of AdipoR1 expression observed in the present study remains uncertain. However, experimental studies have shown that APN inhibits the growth of OC cells and that increased AdipoR1 and AdipoR2 expression enhances cellular responsiveness to APN [59]. Likewise, Tiwari et al. proposed that APN signaling through AdipoR1 and AdipoR2 may influence tumor metabolism and cell proliferation [60]. In our cohort, however, circulating APN concentrations were not associated with the expression of either receptor, indicating that no direct association between circulating APN concentrations and receptor expression was detected in the present study. Regarding the LPN receptor, LepR positivity was observed in 37.1% of malignant ovarian tumors and 23.1% of benign tumors, without significant differences between groups. This frequency was lower than that reported in previous studies. Uddin et al. evaluated 156 EOC specimens and reported overexpression of LPN receptors in 59.2% of cases. Moreover, LepR overexpression was identified as an independent predictor of poorer progression-free survival [61]. In a Mexican cohort, Méndez et al. reported positive LepR immunostaining in 75% of OC specimens [62]. Finally, no significant associations were observed between circulating APN or LPN concentrations and their corresponding receptors. Likewise, receptor expression was not associated with any anthropometric or body composition variables. These findings do not exclude biological interactions between ADPs and their receptors, but suggest that such relationships were not detectable in the present cohort.

Although the findings of this study provide novel insights into the associations between circulating ADPs, receptor expression, body composition, and EOC, they should be interpreted within the context of certain limitations. First, the cross-sectional design does not allow the establishment of temporal or causal relationships between the evaluated variables and disease status. Therefore, prospective studies are needed to determine the potential clinical utility of these molecules throughout the course of the disease. Second, visceral fat was estimated using bioelectrical impedance analysis, a practical and widely used method in clinical settings; however, factors such as hydration status and the presence of ascites or large pelvic masses may influence body composition measurements. Although weight and body composition assessments were repeated after surgery in patients with documented ascites to minimize this potential source of bias, some measurement variability cannot be completely excluded. Third, IHC analyses were performed in the subset of cases with available tissue samples, which may have influenced the representativeness of the tissue-based findings. In addition, although the study was conducted at a single tertiary referral center serving patients from western Mexico, the sample size available for certain subgroup analyses was limited, which may affect the generalizability of some findings. The proportional odds assumption was not fully met in the ordinal logistic regression analyses; therefore, a partial proportional odds model was performed as a sensitivity analysis, showing that the associations varied across cumulative outcome thresholds. Furthermore, given the growing number of clinical, metabolic, inflammatory, and treatment-related factors that have been reported to influence ADP regulation and signaling, not all potentially relevant variables could be incorporated into the present study; therefore, residual confounding from unmeasured or unaccounted factors cannot be excluded. Several correlation analyses were exploratory and involved multiple comparisons among ADPs, nutritional variables, and clinical biomarkers. Therefore, some of the statistically significant associations, particularly those of modest magnitude, may represent chance findings and should be interpreted cautiously and considered hypothesis-generating until confirmed in independent cohorts. Future studies incorporating larger multicenter cohorts with longitudinal follow-up and a broader assessment of metabolic and inflammatory pathways are warranted to further clarify the interplay and clinical relevance of ADPs, receptor expression, and nutritional status in EOC, as well as their potential clinical and prognostic relevance.

## 5. Conclusions

This study provides a comprehensive evaluation of circulating ADPs, tissue receptor expression, body composition, dietary intake, and clinicopathological characteristics in women with benign ovarian tumors and EOC. Among the ADPs evaluated, LPN and RST emerged as the most relevant findings in relation to disease status. Lower serum LPN levels and higher serum RST levels were observed in women with EOC and remained independently associated with higher categories within the predefined clinical ordering after adjustment for visceral fat, age, and menopausal status. Lower LPN concentrations were also independently associated with advanced-stage disease in the EOC-only analysis. Among the nutritional variables evaluated, visceral fat was independently associated with higher categories within the predefined clinical ordering, although this association varied across cumulative outcome thresholds. In addition, AdipoR1 expression was significantly more frequent in malignant than in benign ovarian tumors. Together, these findings provide a more comprehensive characterization of circulating ADPs, body composition, and receptor expression in relation to EOC and disease status and identify LPN, RST, and visceral fat as variables warranting further investigation. Nevertheless, further prospective and mechanistic studies are needed to elucidate the biological mechanisms underlying the reduction in circulating LPN concentrations, the increase in circulating RST concentrations, and the regulation of APN receptor expression in EOC, as well as to determine their potential clinical relevance.

## Figures and Tables

**Table 1:** Clinical characteristics of the study participants.

	Controls (n = 19)	Benign Tumors (n = 89)	Malignant Tumors (n = 43)	*p*
Age (years)	42.5 ± 11.2 ^3^	42.7 ± 12.8 ^3^	51.7 ± 7.9 ^1,2^	<0.0001
Age at menarche	12.1 ± 1.5	12.2 ± 1.7	12.1 ± 1.7	0.870
Parity	2 (0.5–2.5)	2 (0–3)	2 (1–3)	0.720
CA125 (U/mL)	20.2 (17.2–22.8) ^3^	51.6 (17.7–196.9) ^3^	661.6 (256.1–778.1) ^1,2^	<0.0001
HE4 (pg/mL)	1987 (1821.0–2154.0) ^3^	2796 (2220.0–3520.0) ^3^	5086 (3910.0–5726.0) ^1,2^	<0.0001
ROMA index (%)	18.9 (13.0–25.1) ^3^	30.6 (20.1–45.0) ^3^	86.5 (68.7–90.7) ^1,2^	<0.0001
Menopausal status				<0.001
No	13 (68.4%)	63 (70.7%) ^3^	14 (32.5%) ^2^	
Yes	6 (31.6%)	26 (29.2%) ^3^	29 (67.4%) ^2^	
History of oral contraceptive use				0.902
No	6 (31.5%)	31 (34.8%)	16 (37.2%)	
Yes	13 (68.4%)	53 (59.5%)	26 (60.4%)	
Not reported	-	5 (5.6%)	1 (2.3%)	
Smoking status				0.868
No	15 (78.9%)	63 (70.7%)	34 (79.0%)	
Yes	4 (21.1%)	22 (24.7%)	8 (18.6%)	
Not reported	-	4 (4.4%)	1 (2.3%)	
Diabetes mellitus				0.208
No	19 (100%)	77 (86.5%)	36 (83.7%)	
Yes	0 (0%)	12 (13.5%)	7 (16.3%)	

Data are presented as mean ± standard deviation (SD) or median (interquartile range, IQR), according to the distribution of each variable, for continuous variables and as frequency (percentage) for categorical variables. Superscript numbers indicate statistically significant differences between groups, where ^1^ corresponds to the control group, ^2^ to the benign tumor group, and ^3^ to the malignant tumor group. Statistical analyses were performed using the Kruskal–Wallis test, Welch’s ANOVA, chi-square test, or Fisher’s exact test, as appropriate according to variable type and distribution. Post hoc comparisons were conducted using the Games–Howell test following Welch’s ANOVA or Dunn’s test with Bonferroni correction following the Kruskal–Wallis test, as appropriate. The *p*-value represents the overall comparison among the three groups.

**Table 2:** Tumor Characteristics of Patients with Ovarian Tumors.

	Benign Tumors (n = 89)	Malignant Tumors (n = 43)	*p*
Tumor size (cm)	7.5 (5.8–11.0)	11.7 (7.8–16.4)	<0.01
Histological subtype	Fibroma, 1 (1.1%) Brenner Tumor, 1 (1.1%) Mucinous Cystadenoma, 8 (9.0%) Ovarian Cyst, 11 (12.4%) Mature Teratoma, 15 (16.9%) Serous Cystadenoma, 20 (22.5%) Endometrioma, 33 (37.0%)	Low-Grade Serous Carcinoma, 4 (9.3%) Mucinous Carcinoma, 6 (13.9%) Clear Cell Carcinoma, 8 (18.6%) Endometrioid Carcinoma, 9 (20.9%) High-Grade Serous Carcinoma, 16 (37.2%)	NA
FIGO stage			NA
Stage I	-	17 (39.5%)	
Stage II	-	3 (6.9%)	
Stage III	-	18 (41.8%)	
Stage IV	-	3 (6.9%)	
Not Reported	-	2 (4.6%)	

Data are presented as mean ± standard deviation (SD) or median (interquartile range, IQR), according to the distribution of each variable, and as frequency (percentage) for categorical variables. Tumor size was compared using the Mann–Whitney U test. Histological subtype and FIGO stage are presented descriptively; therefore, no statistical comparisons were performed. NA = not applicable.

**Table 3:** Components of Nutritional Status.

	Controls (n = 19)	Benign Tumors (n = 89)	Malignant Tumors (n = 43)	*p*
Anthropometric and Body Composition Variables				
BMI (kg/m^2^)	24.8 ± 2.8 ^2,3^	28.8 ± 5.5 ^1^	27.9 ± 5.2 ^1^	<0.0001
MUAC (cm)	28.5 ± 2.8	29.5 ± 4.3	27.6 ± 4.5	0.094
Total body fat (%)	35.2 ± 4.6	38.6 ± 7.8	37.1 ± 9.8	0.065
Muscle mass (kg)	39.4 ± 4.0	42.1 ± 6.7	39.4 ± 8.7	0.056
Visceral fat index	5.7 ± 2.2 ^2,3^	7.8 ± 3.5 ^1^	8.2 ± 3.1 ^1^	<0.01
Dietary Intake				
Total energy intake (kcal/day)	1318 ± 409.3	1504 ± 398.1	1328 ± 455.9	0.054
Carbohydrates (% energy)	50.9 ± 9.6	54.9 ± 9.2	53.9 ± 13.3	0.270
Lipids (% energy)	27.0 ± 8.0	24.1 ± 7.0	25.6 ± 9.9	0.319
Protein (% energy)	21.3 ± 5.1	20.7 ± 5.8	20.3 ± 6.2	0.755
Saturated fatty acids (g/day)	2.1 (1.0–3.7) ^2,3^	0.8 (0.4–1.7) ^1^	0.4 (0.4–2.6) ^1^	<0.05
Folate (μg/day)	152.5 (101.7–188.3) ^2,3^	71.4 (33.7–142.6) ^1^	63.3 (38.3–107.8) ^1^	<0.01
Iron (mg/day)	6.5 (3.9–9.0) ^2^	8.8 (6.8–11.4) ^1^	7.2 (5.2–10.4)	<0.05
Non-heme iron (mg/day)	1.4 (1.0–2.1) ^2,3^	0.8 (0.5–1.3) ^1^	1.0 (0.3–1.5) ^1^	<0.05
Metabolic Variable				
Serum Glucose (mg/dL)	98.0 (90.0–103.0)	95.5 (87.0–109.0)	101.0 (89.2–113.7)	0.448
Nutrition-Related Symptoms				
Self-reported early satiety				<0.0001
No	19 (100%) ^2,3^	41 (46.1%) ^1^	17 (39.5%) ^1^	
Yes	0 (0%)	41 (46.1%)	24 (55.8%)	
Not reported	0 (0%)	7 (7.8%)	2 (4.6%)	

Data are presented as the mean ± standard deviation (SD) or median (interquartile range, IQR), according to the distribution of each variable, for continuous variables and as the frequency (percentage) for categorical variables. Superscript numbers indicate statistically significant differences between groups, where ^1^ corresponds to the control group, ^2^ to the benign tumor group, and ^3^ to the malignant tumor group. Statistical analyses were performed using the Kruskal–Wallis test, Welch’s ANOVA, one-way ANOVA, chi-square test, or Fisher’s exact test, as appropriate, according to the variable type and distribution. Post hoc comparisons were conducted using Dunn’s test with Bonferroni correction or the Games–Howell test, as appropriate. The *p*-value represents the overall comparison among the three groups.

**Table 4:** Expression of adipokine receptors.

	Malignant (n = 35)	Benign (n = 52)	*p*
AdipoR1			<0.01
Negative	4 (11.4%)	20 (38.5%)	
Positive	31 (88.6%)	32 (61.5%)	
AdipoR2			0.646
Negative	34 (97.1%)	49 (94.2%)	
Positive	1 (2.9%)	3 (5.8%)	
LepR			0.227
Negative	22 (62.9%)	40 (76.9%)	
Positive	13 (37.1%)	12 (23.1%)	

Data are presented as frequency (percentage). Statistical comparisons between malignant and benign ovarian tissues were performed using Fisher’s exact test or the chi-square test, as appropriate.

## Data Availability

The original contributions presented in this study are included in the article. Further inquiries can be directed to the corresponding authors.

## Supplementary Materials

The following supporting information can be downloaded at: https://www.mdpi.com/article/10.3390/cancers18162712/s1.
