# Supplementary material for: Circulating Adipokines, Tissue Receptor Expression, and Body Composition: Clinical Relevance and Disease Stage in Epithelial Ovarian Carcinoma
Source: Cancers (Basel). 2026 Aug 21;18(16):2712. doi: 10.3390/cancers18162712 (PMC13511053; doi:10.3390/cancers18162712)
Supplement: Supplementary file 1 [file cancers-18-02712-s001.zip › Supplementary Figure S1_Additional_IHC_Images.pdf]

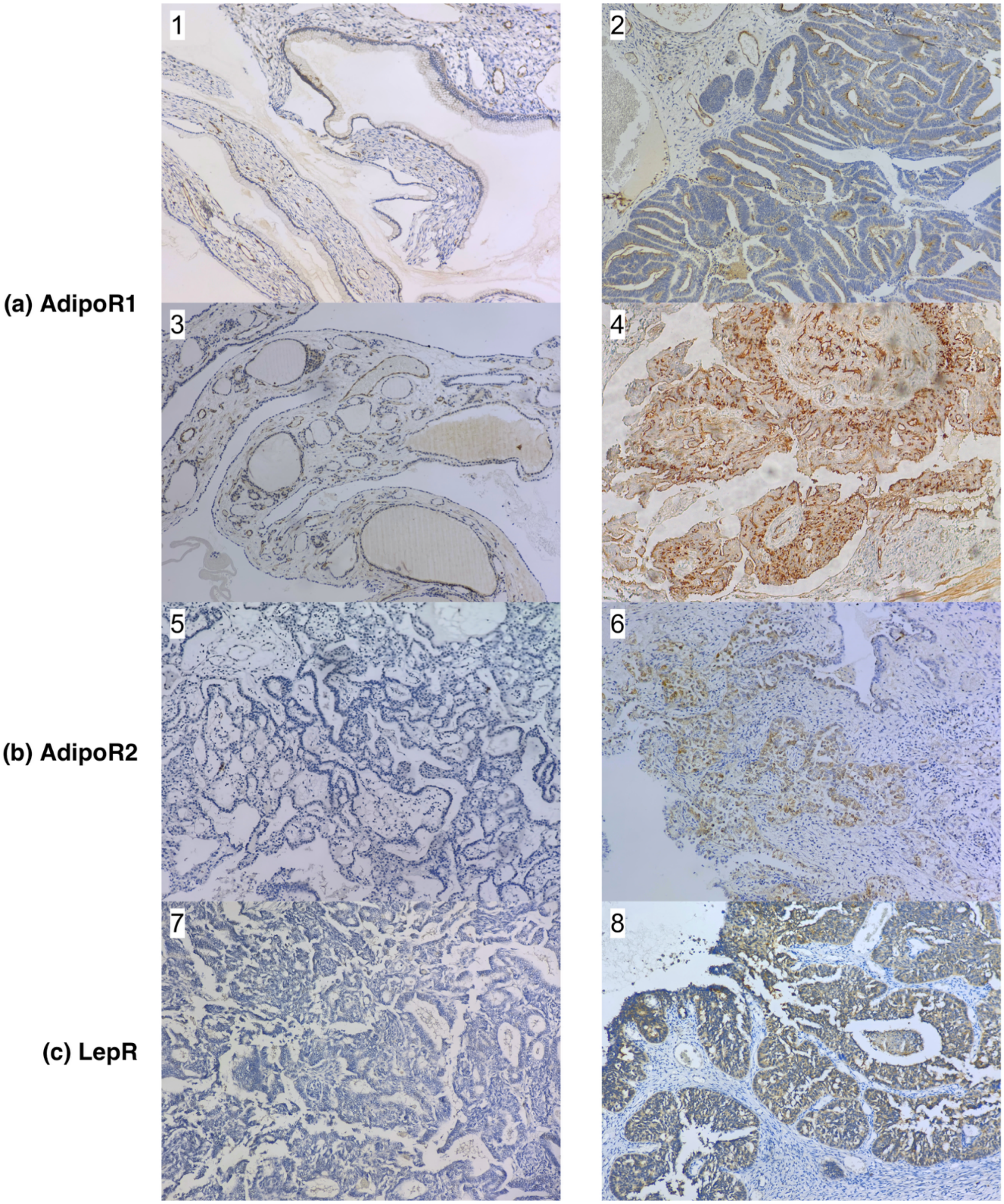

Supplementary Figure S1. Additional representative immunohistochemical images illustrating the variability in adipokine receptor expression across benign and malignant ovarian tumors. (a) Additional representative AdipoR1 staining patterns showing (1) negative expression in a mucinous cystadenoma, (2) positive expression in an endometrioid carcinoma, (3) negative expression in a mature teratoma, and (4) positive expression in a low-grade serous carcinoma. (b) Additional representative AdipoR2 staining patterns showing

negative expression in (5) an endometrioid carcinoma and (6) a clear cell carcinoma. (c) Additional representative LepR staining patterns showing (7) negative expression in an endometrioid carcinoma and (8) positive expression in a low-grade serous carcinoma. All images were acquired using a 10× objective.
