# Supplementary material for: Circulating Adipokines, Tissue Receptor Expression, and Body Composition: Clinical Relevance and Disease Stage in Epithelial Ovarian Carcinoma
Source: Cancers (Basel). 2026 Aug 21;18(16):2712. doi: 10.3390/cancers18162712 (PMC13511053; doi:10.3390/cancers18162712)
Supplement: Supplementary file 1 [file cancers-18-02712-s001.zip › Supplementary Table S1. Dietary intake assessment of the study population.pdf]

Supplementary Table S1. Dietary intake assessment of the study population

	Controls (n = 19)	Benign Tumors (n = 89)	Malignant Tumors (n = 43)	p
Polyunsaturated fatty acids (g)	2.3 (1.5-3.0)	1.5 (1.5-3.0)	1.5 (1.4-1.5)	0.055
Monounsaturated fatty acids (g)	5.7 (2.9-7.5)	2.9 (2.9-5.8)	2.9 (2.9-3.3)	0.060
Cholesterol (mg)	182.8 (109.8-428.2)	275.9 (132.7-467.2)	363.9 (164.8-476.6)	0.599
Vitamin A (μg)	501.8 (258.1-707.0)	349.0 (244.8-557.3)	320.4 (209.8-468.0)	0.209
Ascorbic acid (mg)	32.9 (24.6-118.0)	29.8 (9.6-80.8)	40.4 (11.4-86.9)	0.235
Dietary fiber (g)	12.2 (7.8-17.5)	15.1 (11.0-20.8)	13.7 (9.6-20.9)	0.670
Potassium (mg)	552.8 (447.6-964.0)	506.0 (291.0-795.3)	559.7 (280.9-836.3)	0.308
Calcium (mg)	463.7 (277.2-745.0)	607.7 (361.9-808.9)	450.1 (294.2-709.6)	0.071
Selenium (μg)	28.5 (21.3-51.1)	45.5 (34.7-56.5)	45.1 (31.6-55.1)	0.151

Continuous variables are presented as median (interquartile range, IQR). Comparisons among the three study groups were performed using the Kruskal–Wallis test. The reported *p*-values correspond to the overall comparison among the three groups.
