# Supplementary material for: Circulating Adipokines, Tissue Receptor Expression, and Body Composition: Clinical Relevance and Disease Stage in Epithelial Ovarian Carcinoma
Source: Cancers (Basel). 2026 Aug 21;18(16):2712. doi: 10.3390/cancers18162712 (PMC13511053; doi:10.3390/cancers18162712)
Supplement: Supplementary file 1 [file cancers-18-02712-s001.zip › Supplementary Table S2. Effect sizes for Kruskal–Wallis analyses of circulating adipokines according to study group and FIGO category.pdf]

Supplementary Table S2. Effect sizes for Kruskal–Wallis analyses of circulating adipokines according to study group and FIGO category

Adipokine	Comparison	η²[H]	Magnitude
**Leptin**	Study group	0.067	Moderate
**Resistin**	Study group	0.100	Moderate
**Adiponectin**	Study group	0.016	Small
**Adipsin**	Study group	0.000	Small
**Leptin**	FIGO category	0.094	Moderate
**Resistin**	FIGO category	0.087	Moderate
**Adiponectin**	FIGO category	0.017	Small
**Adipsin**	FIGO category	0.030	Small

Effect sizes were estimated using eta-squared based on the Kruskal–Wallis H statistic ($\eta^2[H]$). Effect sizes were interpreted as small (0.01–<0.06), moderate (0.06–<0.14), and large (≥0.14).
