# Supplementary material for: Circulating Adipokines, Tissue Receptor Expression, and Body Composition: Clinical Relevance and Disease Stage in Epithelial Ovarian Carcinoma
Source: Cancers (Basel). 2026 Aug 21;18(16):2712. doi: 10.3390/cancers18162712 (PMC13511053; doi:10.3390/cancers18162712)
Supplement: Supplementary file 1 [file cancers-18-02712-s001.zip › Supplementary Table S3. Spearman correlations between age and circulating adipokine concentrations.pdf]

Supplementary Table S3. Spearman correlations between age and circulating adipokine concentrations

Adipokine	Overall cohort (ϱ)	p	EOC only (ϱ)	p
**Adipsin**	0.21	< 0.05	0.06	0.713
**Adiponectin**	0.10	0.216	0.35	< 0.05
**Leptin**	-0.13	0.113	-0.30	<0.05
**Resistin**	0.08	0.356	-0.06	0.697

Spearman rank correlation coefficients were calculated to assess the relationship between age and circulating adipokine concentrations in the overall study population and among women with epithelial ovarian cancer (EOC). Statistically significant correlations are shown in bold. ϱ, Spearman correlation coefficient; EOC, epithelial ovarian cancer
