# Supplementary material for: Circulating Adipokines, Tissue Receptor Expression, and Body Composition: Clinical Relevance and Disease Stage in Epithelial Ovarian Carcinoma
Source: Cancers (Basel). 2026 Aug 21;18(16):2712. doi: 10.3390/cancers18162712 (PMC13511053; doi:10.3390/cancers18162712)
Supplement: Supplementary file 1 [file cancers-18-02712-s001.zip › Supplementary Table S4. Sensitivity analysis of the ordinal logistic regression model.pdf]

Supplementary Table S4. Sensitivity analysis of the ordinal logistic regression model

Variable	OR	95% CI	p
**Model A. Adjusted for Visceral fat, age, and menopausal status**			
Adipsin, per SD increase	0.97	0.63-1.47	0.899
Adiponectin, per SD increase	0.83	0.56-1.24	0.373
Leptin, per SD increase	0.45	0.27-0.74	< 0.01
Resistin, per SD increase	2.24	1.53-3.36	< 0.001
Visceral fat, per SD increase	2.58	1.35-5.08	< 0.01
Age, per SD increase	0.71	0.35-1.42	0.329
Menopausal status, Yes vs. No	2.82	0.91-9.02	0.076
**Model B. Adjusted for BMI, age, and menopausal status**			
Adipsin, per SD increase	0.99	0.66-1.48	0.980
Adiponectin, per SD increase	0.85	0.58-1.25	0.416
Leptin, per SD increase	0.50	0.31-0.79	< 0.01
Resistin, per SD increase	1.92	1.35-2.77	< 0.001
BMI, per SD increase	1.98	1.21-3.29	< 0.01
Age, per SD increase	1.20	0.70-2.08	0.507
Menopausal status, Yes vs. No	2.17	0.74-6.51	0.163
**Model C. Partial proportional odds model (sensitivity analysis)**			
**Variable**	**Cumulative threshold**	**OR (95% CI)**	**p**
**Threshold-specific effects**			
Leptin	Control ǀ Benign	0.74 (0.32-1.68)	0.467
	Benign ǀ Early EOC	0.16 (0.06-0.41)	<0.001
	Early EOC ǀ Advanced EOC	0.12 (0.04-0.41)	<0.001
Resistin	Control ǀ Benign	9.55 (2.12-43.08)	< 0.01
	Benign ǀ Early EOC	1.84 (1.17-2.89)	< 0.01
	Early EOC ǀ Advanced EOC	1.26 (0.76-2.08)	0.372
Visceral fat	Control ǀ Benign	2.92 (1.16-7.36)	< 0.05
	Benign ǀ Early EOC	3.70 (1.59-8.58)	< 0.01

	Early EOC ǀ Advanced EOC	1.28 (0.50–3.25)	0.608
**Proportional effects**			
Adipsin	-	1.07 (0.70–1.64)	0.765
Adiponectin	-	0.74 (0.49–1.13)	0.159
Age	-	0.64 (0.30–1.35)	0.242
Menopausal status	-	2.55 (0.80–8.13)	0.115

The ordinal outcome was defined as Control < Benign ovarian tumor < Early-stage epithelial ovarian cancer (EOC) < Advanced-stage EOC. Continuous variables were standardized prior to analysis; therefore, odds ratios (ORs) represent the change in odds associated with a one-standard-deviation increase in each variable. Premenopausal women were used as the reference category for menopausal status. A partial proportional odds model was additionally fitted as a sensitivity analysis. Leptin, resistin, and visceral fat were modeled with threshold-specific effects, whereas adipsin, adiponectin, age, and menopausal status retained proportional effects. The cumulative thresholds correspond to Control vs. Benign/Early EOC/Advanced EOC, Control/Benign vs. Early/Advanced EOC, and Control/Benign/Early EOC vs. Advanced EOC, respectively. OR, odds ratio; CI, confidence interval; SD, standard deviation; EOC, epithelial ovarian carcinoma.
