# Supplementary material for: Circulating Adipokines, Tissue Receptor Expression, and Body Composition: Clinical Relevance and Disease Stage in Epithelial Ovarian Carcinoma
Source: Cancers (Basel). 2026 Aug 21;18(16):2712. doi: 10.3390/cancers18162712 (PMC13511053; doi:10.3390/cancers18162712)
Supplement: Supplementary file 1 [file cancers-18-02712-s001.zip › Supplementary Table S5. Variance inflation factors (VIFs).pdf]

Supplementary Table S5. Variance inflation factors (VIFs)

Variable	Primary adjusted ordinal logistic regression	Binary logistic regression (Benign vs Malignant)
Adipsin	1.37	1.29
Adiponectin	1.21	1.23
Leptin	1.94	2.12
Resistin	1.13	1.29
Visceral fat	3.17	3.33
Age	4.10	3.58
Menopausal status	2.58	2.46

Variance inflation factors (VIFs) were calculated to assess multicollinearity among predictors included in the final adjusted ordinal logistic regression model. All VIF values were below the commonly used threshold of 5, indicating acceptable levels of multicollinearity among the retained predictors.
