# Supplementary material for: Circulating Adipokines, Tissue Receptor Expression, and Body Composition: Clinical Relevance and Disease Stage in Epithelial Ovarian Carcinoma
Source: Cancers (Basel). 2026 Aug 21;18(16):2712. doi: 10.3390/cancers18162712 (PMC13511053; doi:10.3390/cancers18162712)
Supplement: Supplementary file 1 [file cancers-18-02712-s001.zip › Supplementary Table S6. Numerical results of the binary logistic regression models presented in Figure 6.pdf]

Supplementary Table S6. Numerical results of the binary logistic regression models presented in Figure 6.

(A) Binary logistic regression model discriminating benign from malignant ovarian tumors		
**Variable**	**OR (95% CI)**	**p**
Adipsin	0.94 (0.57-1.53)	0.789
Adiponectin	0.98 (0.60-1.58)	0.947
Leptin	0.47 (0.21-0.91)	< 0.05
Resistin	1.78 (1.11-2.95)	< 0.05
Visceral fat	1.56 (0.69-3.60)	0.288
Age	0.93 (0.39-2.27)	0.879
Menopausal status	4.17 (1.03-18.13)	< 0.05
**(B) Binary logistic regression models discriminating advanced versus early stage EOC**		
**Adipsin model**		
Adipsin	0.58 (0.27-1.13)	0.127
Age	0.60 (0.20-1.73)	0.354
Menopausal status	3.28 (0.36-34.96)	0.298
**Adiponectin model**		
Adiponectin	1.73 (0.86-3.93)	0.147
Age	0.45 (0.14-1.29)	0.149
Menopausal status	3.85 (0.43-40.82)	0.237
**Leptin model**		
Leptin	0.44 (0.19-0.94)	< 0.05
Age	0.40 (0.12-1.18)	0.113
Menopausal status	3.25 (0.34-34.75)	0.308
**Resistin model**		
Resistin	1.19 (0.62-2.38)	0.604
Age	0.54 (0.18-1.49)	0.248
Menopausal status	4.02 (0.45-43.96)	0.226

Panel (A) shows a multivariable binary logistic regression model adjusted for visceral fat, age, and menopausal status. In panel (B), separate multivariable binary logistic regression models were fitted for each adipokine, each adjusted for age and menopausal status. OR, odds ratio; CI, confidence interval; EOC, epithelial ovarian carcinoma. Continuous predictors were standardized prior to analysis; therefore, ORs are expressed per one-standard-deviation increase.
